# CoNet: Efficient Network Regression for Survival Analysis in Transcriptome-Wide Association Studies—With Applications to Studies of Breast Cancer

**DOI:** 10.3390/genes14030586

**Published:** 2023-02-25

**Authors:** Jiayi Han, Liye Zhang, Ran Yan, Tao Ju, Xiuyuan Jin, Shukang Wang, Zhongshang Yuan, Jiadong Ji

**Affiliations:** 1 Department of Biostatistics, School of Public Health, Cheeloo College of Medicine, Shandong University, Jinan 250012, China; 2 Institute for Medical Dataology, Shandong University, Jinan 250003, China; 3 Institute for Financial Studies, Shandong University, Jinan 250100, China

**Keywords:** TWAS, biological network, breast cancer, survival analysis

## Abstract

Transcriptome-wide association studies (TWASs) aim to detect associations between genetically predicted gene expression and complex diseases or traits through integrating genome-wide association studies (GWASs) and expression quantitative trait loci (eQTL) mapping studies. Most current TWAS methods analyze one gene at a time, ignoring the correlations between multiple genes. Few of the existing TWAS methods focus on survival outcomes. Here, we propose a novel method, namely a COx proportional hazards model for NEtwork regression in TWAS (CoNet), that is applicable for identifying the association between one given network and the survival time. CoNet considers the general relationship among the predicted gene expression as edges of the network and quantifies it through pointwise mutual information (PMI), which is under a two-stage TWAS. Extensive simulation studies illustrate that CoNet can not only achieve type I error calibration control in testing both the node effect and edge effect, but it can also gain more power compared with currently available methods. In addition, it demonstrates superior performance in real data application, namely utilizing the breast cancer survival data of UK Biobank. CoNet effectively accounts for network structure and can simultaneously identify the potential effecting nodes and edges that are related to survival outcomes in TWAS.

## 1. Introduction

Genome-wide association studies (GWASs) have detected hundreds of thousands of single nucleotide polymorphisms (SNPs) that are related with complex diseases, including various cancers [1]. However, most GWAS signals are located in non-coding regions across the genome [2], leading to difficulties in the validation and interpretation of associations, and challenges in uncovering the regulatory mechanism underlying the disease. Concurrently, expression quantitative trait loci (eQTL) mapping studies have successfully detected several genetic variants that are related to gene expression. By integrating GWAS and eQTL studies, the recently developed transcriptome-wide association study (TWAS) provides a promising technique for interpreting GWAS associations and identifying disease-related genes. TWAS has facilitated the identification of potential genes that have expression values associated with various GWAS outcome traits, such as lung cancer [3], pancreatic cancer [4], and schizophrenia [5]. In typical TWAS analysis, the effect size of the genotype on gene expression is first estimated from the eQTL study, which is further used to predict gene expression in GWAS. Then, the regression association analysis is usually conducted between the gene expression prediction and the trait in GWAS. To date, many TWAS statistical tools have been developed; some methods aim to improve the performance of genotype effect size estimation using different models (e.g., PrediXcan [6], TWAS [7], DPR [8], and TIGAR [9]), some aim to improve the statistical power in the final association analysis of TWAS (e.g., kernel-type methods [10]), and some make the statistical inference in a likelihood framework to account for the uncertainty in the estimation of the genotype effect size (e.g., PMR-Egger [11] and moPMR-Egger [12]). 

It should be noted that most currently available TWAS methods encounter important challenges. The first is that most current TWAS analyses can only focus on one gene at a time, thus ignoring the correlation structure among multiple genes. To the best of our knowledge, FOCUS [13] and FOGS [14] are the only two existing multiple gene-based TWAS methods. FOCUS constructs the multiple-gene TWAS model from a Bayesian perspective, aiming to obtain credible gene sets that contain all the associated genes at a nominal confidence level. FOGS is essentially a multiple SNP model, which identifies genes based on conditional analyses of SNPs of each gene by adjusting the other SNPs residing in the same region. However, both methods fail to take the network relationship among multiple genes into account, thus resulting in these methods possibly losing efficiency. 

A complex disease can reflect the interactions among multiple genes in a biological network [15]. Identifying the specific biological network related with a complex disease can help explore the network mechanism of complex diseases. Often, in a multiple-gene network, nodes are used to represent genes and edges are used to represent the possible interactions between different genes. Correspondingly, genes and their interactions included in the network can make contributions to the development of disease. Quantifying the correlation between nodes to represent the edge is challenging: it is not easy to determine the suitable measure to capture the general between-node correlations. PMI was confirmed as an efficient measure to represent the complex relationship among different network nodes in the network regression [16].

Previously, we have developed two network regression method in TWAS: the NeRiT [17] method for continuous outcomes and PoLoNet [18] for binary or categorical outcomes, which illustrated the advantage of PMI in capturing the general relationship among different network nodes. However, these methods cannot be easily extended to the survival outcomes context given that the distribution of the survival time is often non-normal, coupled with the censoring issue. On the other hand, time-to-event data are commonly encountered in GWAS, especially in cancer genomics [19,20]. For example, in the UK Biobank, the breast cancer survival time is often the main outcome for exploring the biological network related to breast cancer progression, but some participants may be censored and are unable to experience the event by the end of the follow-up. 

In this study, a COx proportional hazards model for NEtwork regression in TWAS (CoNet) was developed to detect the association between one given network and the survival time. CoNet is developed under a two-stage TWAS framework. In the first stage, the SNP effect size for each specific gene within one network is estimated in the eQTL study. In the second stage, CoNet adopts PMI to quantify the edges of the network to describe the general relationship among the nodes and conducts the association analysis with all the nodes along with all the edges in the model. With the network structure effectively accounted for, CoNet can simultaneously identify the potential nodes and edges that are associated with the survival time. Comprehensive and extensive realistic simulations are conducted to evaluate the performance of CoNet, including the type I error control and power for detecting either the node effect or the edge effect. In addition, breast cancer survival data in UK Biobank were used to highlight the advantages of CoNet in real applications.

## 2. Materials and Methods

### 2.1. eQTL Study

Suppose there are a total of *m* genes and $x_{i}$ is an $n_{1}$-vector of gene expression measurements for the *i*-th gene, which is measured on $n_{1}$ individuals in the gene expression study. $G_{x_{i}}$ is denoted as an $n_{1}$ by $p_{i}$ matrix of genotypes for $p_{i}$ cis-SNPs (1 Mb windows around the gene) of the *i*-th gene. In two-stage TWAS, it is common to obtain the genotype effect size using the following model:

(1) $$x_{i}=G_{x_{i}}\beta_{i}+\varepsilon_{x_{i}},\left(i=1,2,\ldots ,m\right)$$

where $\beta_{i}$ is a $p_{i}$-vector of cis-SNP effect sizes on the *i*-th gene expression and $\;\varepsilon_{x_{i}}$ is an error term with $n_{1}$ sampled values that is distributed from a normal distribution $N\left(0,\sigma_{x}^{2}\right)$. 

### 2.2. Gene Expression Prediction

The estimator of genotype effect size ${\hat{\beta }}_{i}\;$ can be obtained from the eQTL study and the predicted gene expression of the *i*-th gene is derived as ${\tilde{x}}_{i}=G_{yi}\;{\hat{\beta }}_{i}$, where $G_{y_{i}}\;$ is the $n_{2}$ by $p_{i}$ matrix of genotypes for the same $p_{i}$ cis-SNPs of the *i*-th gene measured on $n_{2}\;$ individuals in the GWAS study.

CoNet aims to detect the association between a given network and the survival time under the two-stage TWAS framework, with the prior known network structure. The performance of TWAS depends on the estimation accuracy of the effect of cis-SNP on the gene expression [21] which is strongly associated with the extent of the consistency between the assumed prior distribution and the true distribution of the genetic effect size. However, it is usually hard to access the true distribution of SNP effect size. As BSLMM and DPR modeling assumptions are more flexible than the normality hypothesis and tend to outperform sparse models in predicting gene expression in TWAS applications [22]. Here, we choose the non-parametric Dirichlet process regression (DPR) [8] to model the SNP effect size due to its robustness to the distribution of the genetic effect size. Furthermore, the Bayesian sparse linear mixed model (BSLMM) [23] was also applied for sensitivity analysis. BSLMM is a hybrid modeling assumption between a sparse modeling assumption and the standard polygenic modeling assumption. The SNP effect size is assumed to follow a mixture of two normal distributions in the BSLMM model. We compare the performance of CoNet when using the DPR model in the first stage and when using the BSLMM model in the first stage. Evaluation of the performance of CoNet cannot be substantially influenced by the model assumption of the genetic effect size.

For subject *j* in the GWAS study, $j=1,\ldots ,n_{2}$, the CoNet model based on the Cox Proportional Hazard Model is defined as:

(2) $$h\left(t;Z_{ij},{\tilde{x}}_{ij},E_{jlk}\right)=h_{0}\left(t\right)exp\left(\mu_{0}+\overset{s}{\underset{i=1}{\sum }}Z_{ij}\alpha_{i}+\overset{m}{\underset{i=1}{\sum }}{\tilde{x}}_{ij}\eta_{i}+\overset{m}{\underset{l=1}{\sum }}\overset{m}{\underset{k>l}{\sum }}I_{lk}E_{jlk}\gamma_{lk}\right)$$

where

$$I_{lk}=\{\begin{array}{cc} 1 & l-th\;gene\;and\;k-th\;gene\;are\;connected\;in\;the\;network\\ 0 & otherwise \end{array}$$

and $h_{0}\left(t\right)$ is the baseline hazard function, ${\tilde{x}}_{ij}\;$ denotes the predicted gene expression of the *i*-th gene derived from the above model (4),$\;E_{jlk}$ is the estimator of PMI between the *l*-th and *k*-th node estimated from the BKDE method for the *j*-th individual, $Z_{j}={\left(Z_{1j},Z_{2j},\ldots ,Z_{sj}\right)}^{T}\;$ denotes an *s*-vector of the covariates for the *j*-th individual (e.g., top five genotype principle components (PCs)), $\alpha_{i}\left(i=1,\ldots ,s\right)$ represents the coefficient of the corresponding covariate, $\eta_{i}$(*i* = 1, …, *m*) is the effect of the *i*-th node, and $\gamma_{lk}$ indicates the effect of the edge linking the *l*-th and the *k*-th node. 

CoNet obtains the predicted gene expression included in the target network with the DPR model, then uses PMI to describe the correlations between nodes. After directly plugging the predicted gene expression and PMI estimator among different network nodes, CoNet is regarded as a Cox proportional model. The main goal is to estimate and test the node effects η and edge effects γ. The partial likelihood framework can be utilized to infer the parameters of interest and obtain estimates of $\hat{\eta \;}$ and $\hat{\gamma }$, as well as their standard error values $se\left(\hat{\eta }\right)$ and $se\left(\hat{\gamma }\right)$. Subsequently, the corresponding Wald test can be constructed to obtain a *p*-value for hypothesis testing. CoNet is computationally scalable and implemented in an R package, which is available at https://github.com/hanjiayi626/CoNet (accessed on 20 April 2022).

### 2.3. PMI between Two Predicted Gene Expressions in TWAS Framework

PMI is commonly defined for discrete variables, whereas here we used it for continuous variables. The PMI between ${\tilde{x}}_{i}$ and ${\tilde{x}}_{j}$ is defined as the log ratio between their joint distribution $p\left({\tilde{x}}_{i},{\tilde{x}}_{j}\right)$ and the product of their marginal distribution $p\left({\tilde{x}}_{i}\right)p\left({\tilde{x}}_{j}\right)$ [24]:

(3) $$PMI\left({\tilde{x}}_{i},{\tilde{x}}_{j}\right)=log\frac{p\left({\tilde{x}}_{i},{\tilde{x}}_{j}\right)}{p\left({\tilde{x}}_{i}\right)p\left({\tilde{x}}_{j}\right)}$$

The marginal distribution $p\left({\tilde{x}}_{i}\right)$ and $p\left({\tilde{x}}_{j}\right)\;$ can be estimated using the kernel density estimation method. The joint distribution $p\left({\tilde{x}}_{i},{\tilde{x}}_{j}\right)$ can be estimated using the bivariate kernel density estimation (BKDE) method, which is non-parametric and robust against the misspecification of data distribution [25,26]. Assume $Z_{i}={\left(X_{i},Y_{i}\right)}^{T},i=1,2,\ldots ,n$, is a bivariate sample from a bivariate distribution *p*. The BKDE is:

(4) $${\hat{p}}_{H}\left(z;H\right)=n^{-1}\overset{n}{\underset{i=1}{\sum }}K_{H}\left(z-Z_{i}\right)$$

where $z={\left(x,y\right)}^{T}$ and *H* is the bandwidth (or smoothing) matrix which is symmetric and positive-definite.

$$H=\left(\begin{array}{cc} h_{11} & h_{12}\\ h_{21} & h_{22} \end{array}\right)$$

Bivariate kernel function K is symmetric, with $K_{H}\left(z\right)={\left|H\right|}^{-\frac{1}{2}}K\left(H^{-\frac{1}{2}}z\right)$. This study used the bivariate normal kernel:

(5) $$K_{H}\left(z\right)={\left(2\pi \right)}^{-\frac{d}{2}}{\left|H\right|}^{-\frac{1}{2}}exp\left(-\frac{1}{2}z^{T}H^{-1}z\right)$$

with *d* = 2.

### 2.4. Simulation Study

**Figure 1:**
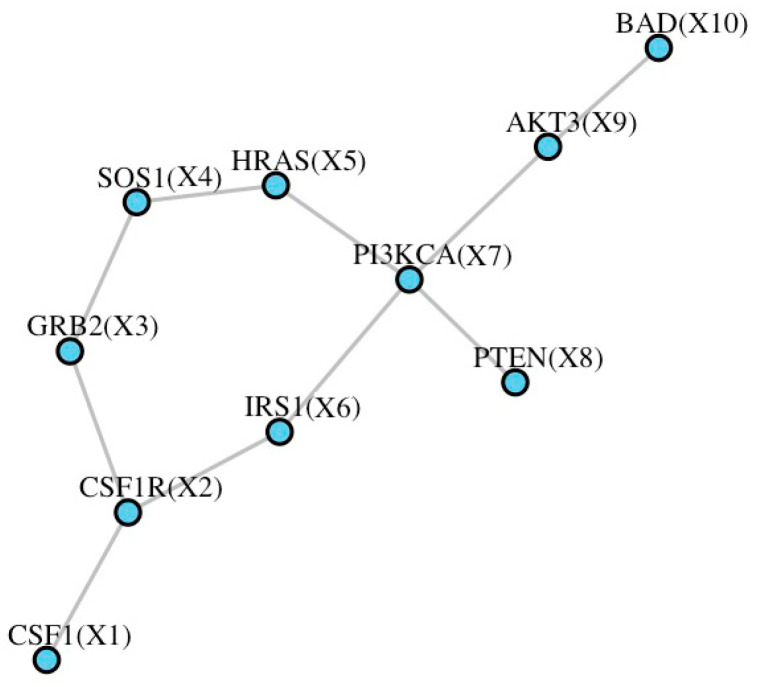
The simulated network for the PI3K-AKT signaling pathway from KEGG.

No other methods have been developed so far for network regression for failure time data in TWAS. Therefore, extensive simulations were performed to compare CoNet with the CPNT method, which was intuitively developed under the CoNet framework but replaced PMI with the product moment (PM) to describe the edges of the network. The expectation of the product of the two scaled node variables (PM) is the linear correlation coefficient, and the PM can be regarded as the individual observed value of the correlation coefficient. To make these simulations more realistic, a realistic TWAS setting was mimicked by integrating data from the GEUVADIS study and GWAS from the UK Biobank. Data from GEUVADIS ($n_{1}=465$) were obtained, then each SNP was standardized along with the gene expression vector (the expression of a specific gene for all the individuals) to obtain a zero mean and a unit standard deviation. For each gene, we chose two models, DPR or BSLMM, to estimate the effects of cis-SNPs on gene expression. The same SNPs were obtained from the UK Biobank, and the genotype vector of each SNP was also standardized. Next, the predicted gene expression was obtained using the standardized genotype matrix. The survival phenotype was also simulated using the above gene expression prediction. Additionally, the PI3K-AKT signaling pathway (hsa04151-nt06214) from the Kyoto Encyclopedia of Genes and Genomes (KEGG) database was selected as the network. All the genes in the pathway overlapped with those in the UK Biobank, containing a total of ten nodes and ten edges (Figure 1). 

For subject *j* (*j* = 1,…,$n_{2})$, according to Bender’s method [27] the complete survival time $T_{j}^{*}\;$ was simulated from a Cox model with a Weibull baseline hazard function as $T_{j}^{*}=-{\left(\frac{log\left(U_{j}\right)}{\lambda exp\left(w_{j}\right)}\right)}^{\frac{1}{v}}$, where the scale parameter *v* was set as 0.5 and shape parameter  λ was set as 1. Furthermore, $U_{j}$ was simulated from a uniform (0,1) distribution and $w_{j}=0.5z_{1j}+0.5z_{2j}+\sum_{i=1}^{m}{\tilde{x}}_{ij}\eta_{i}+\sum_{l=1}^{m}\sum_{k>l}^{m}I_{lk}E_{jlk}\gamma_{lk}$. The covariate $z_{1j}$ was simulated from a standard normal distribution N(0,1) and $z_{2j}$ from a Bernoulli (0.5) distribution. Various GWAS sample sizes ($n_{2}=5000,\;10,000,\;20,000$) were randomly selected from the 337,129 individuals from the UK Biobank. In addition, censoring time $C_{j}$ was randomly simulated on a uniform distribution U (0,$\;T_{j}^{*}$) and $n_{2}q$ censored observations were randomly selected based on the pre-specified censoring rate *q*. Next, the observed time-to-event $T_{j}$ and indicator variables $\delta_{j\;}$ (0 for censored data and 1 for event) were obtained. The censoring rate was set at 10%, 30%, and 50%. The type I error rates were assessed with η=0 and γ=0. Empirical power was evaluated with the effects of genes and edges were set to be η=0.05 and γ=0.05. These effects were calculated to be the 50% quantile from the effect estimate from the real data. Briefly, four scenarios were designed regarding different patterns of the network effect: (1) only nodes of the network have effects (e.g., $X_{6}$); (2) only edges of the network have effects (e.g., $E_{7\_9}$); (3) both nodes and edges have effects, with the nodes hanging on the edges (e.g., $X_{4}$ and $E_{4\_5}$); and (4) both nodes and edges have effects, with the nodes not hanging on the edges (e.g., $X_{2}$ and $E_{4\_5}$). In each scenario, four between-node correlation patterns were considered, including a simple linear correlation, quadratic relationship ($x_{k}=0.1x_{l}{}^{2})$, sine relationship ($x_{k}=sinx_{l}$), and a combination of sine and quadratic relationships ($x_{k}=sin^{2}x_{l}$). For example, if the correlation between node $X_{4}$ and node $X_{5}$ was set to be quadratic, then $X_{5}=0.1X_{4}{}^{2}+\varepsilon$, where ε is the residual error and ε~N(0,1), that is the linear correlation between $X_{4}{}^{2}$ and $X_{5}$, can be used to represent the nonlinear quadratic relationship between $X_{4}$ and $X_{5}$, setting $E_{4\_5}=0.1\cdot X_{4}{}^{2}\cdot X_{5\;}$. In addition to pre-specifying the effecting nodes and effecting edges in each simulation, random selection of the effecting nodes and effecting edges was considered in each simulation to minimize the influence of the network structure. In addition, each setting of the simulations was repeated 1000 times.

### 2.5. Real Data Analysis

CoNet was applied to perform network regression for survival time in a TWAS framework. Specifically, the gene expression data were obtained from the GEUVADIS study, then breast cancer patient survival data from the UK Biobank were examined. The GEUVADIS data [28] include 465 individuals from CEPH (CEU), Finns (FIN), British (GBR), Toscani (TSI), and Yoruba (YRI) populations. Only protein-coding genes and long intergenic non-coding RNAs (lincRNAs) annotated in GENCODE (release 12) [29,30] are considered in this study. Low-expressed genes with zero counts in at least half of the individuals were removed. The PEER normalization method was used to eliminate confounding effects and unwanted variations [8,31]. To remove population stratification, the gene expression measurements were quantile normalized across individuals in each population to a standard normal distribution, which was further quantile normalized to a standard normal distribution across individuals from all five of the populations. A total of 15,810 genes were finally retained. All the individuals also had their genotypes sequenced in the 1000 Genomes Project. Genotype data were therefore obtained from the 1000 Genomes Project phase 3. SNPs with a Hardy–Weinberg equilibrium (HWE) *p*-value < $10^{-4}$, genotype call rate < 95%, or minor allele frequency (MAF) < 0.01 were filtered out. Overall, 7,072,917 SNPS were ultimately left for further analysis.

The UK Biobank data comprises 487,298 individuals and 92,693,895 imputed SNPs [32]. We followed the same sample QC procedure as performed in the Neala laboratory, and 337,129 individuals with European ancestry were retained. SNPs with an HWE *p*-value < $10^{-7}$, genotype call rate < 95%, or MAF < 0.001 were filtered out to retain 13,876,958 SNPs. For each gene, the cis-SNPs that were either within 1Mb upstream of the transcription start site (TSS) or within 1Mb downstream of the transcription end site (TES) were extracted. The cis-SNPs of genes in GEUVADIS were overlapped with those from the UK Biobank to obtain common SNPs. The initial focus was on breast cancer survival and included 818 patients with breast cancer based on the ICD-10 code (C50) within the UK Biobank cohort. Survival time can be calculated by the age of death minus the age at cancer diagnosis. Overall, 241 patients were censored because their time of death was not recorded or there was a competing risk of death. To verify the robustness of the real data, we first searched the networks potentially related to breast cancer from KEGG and involved 7 networks (hsa04630-nt06219, hsa04115, hsa04330, hsa04960, hsa04622, hsa04623, and hsa05211). After overlapping the network genes with those from UK Biobank, we finally analyzed 7 networks, including 338 nodes and 440 edges (Table 1). For both the nodes test and the edges test, we adjusted the p values using the false discover rate (FDR) with the Benjamini-Hochberg (BH) procedure to perform multiple tests, and declared the significance at an FDR threshold of 0.05. In addition, as population stratification may have an impact on the results, the top five PCs were treated as covariates in both the CoNet and the CPNT model.

## 3. Results

### 3.1. Simulation

**Figure 2:**
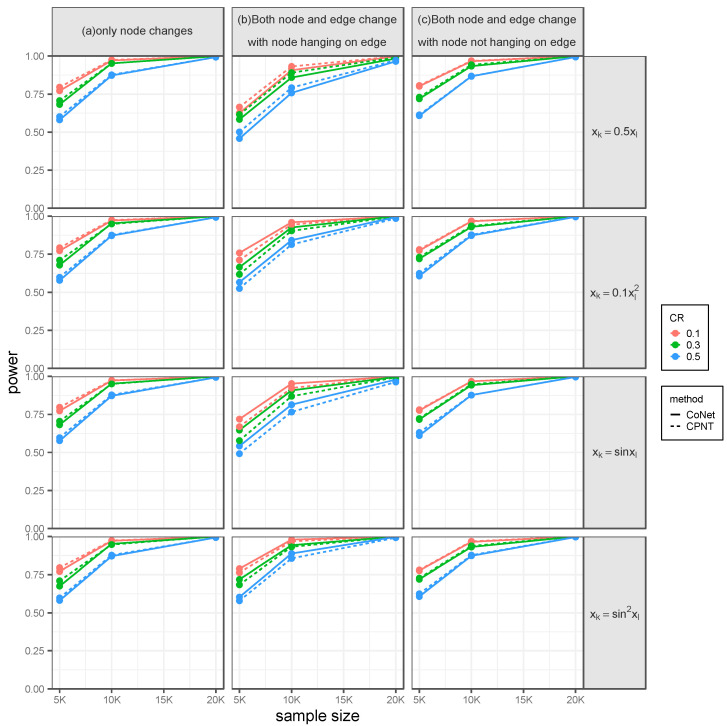
The power for testing the effect of the node on the survival phenotype under the setting that the effecting node is pre-specified, with the SNP effect obtained from the DPR model. Simulations were conducted with four different between-node correlation patterns (the combination of sine and quadratic, sine, quadratic, and linear) and three different censoring rates (0.1, 0.3, and 0.5). (**a**) Only a node has an effect. (**b**) Both node and edge have effects, with the effecting node hanging on the edge. (**c**) Both node and edge have effects, with the effecting node not hanging on the edge.

Since there are currently no statistical tools available for network regression for failure time data in TWAS, here we aimed to compare CoNet with the CPNT method, which was intuitively developed under the CoNet framework but replaced PMI with the PM to describe the network edge. First, when the gene expression was predicted based on the DPR model, both methods performed well in detecting the node effect. Table 2 summarizes the estimated type I error for survival phenotype with a sample size of 5000 under three scenarios. Both methods yielded calibrated type I error control, regardless of the correlation pattern among the network nodes, the censoring rate, or the sample size (Tables S1 and S2). Figure 2 displays the power for the survival phenotype in three scenarios with different node-effecting patterns. Overall, CoNet has a similar power with CPNT: the power of both methods increases as the sample size increases and the censoring rate decreases, regardless of the correlation patterns. 

**Figure 3:**
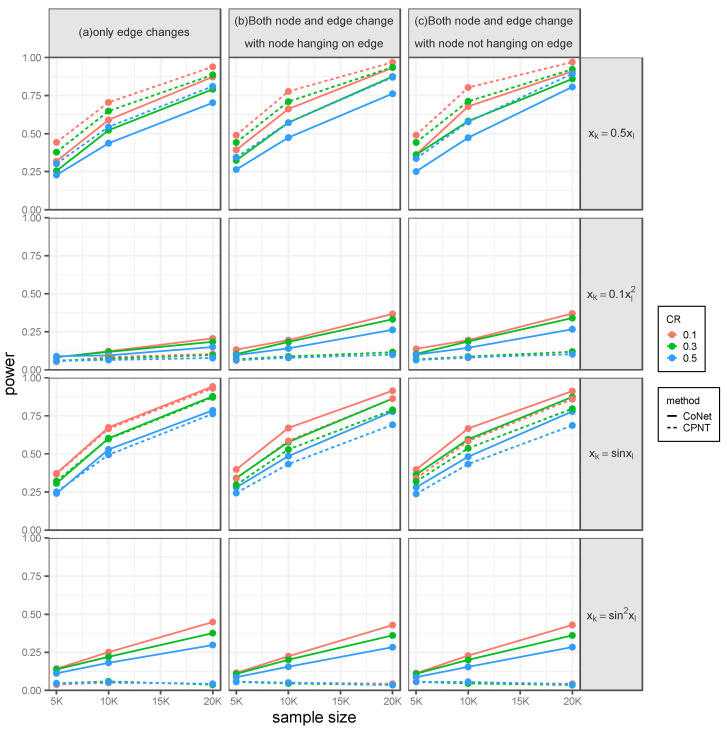
The power for testing the effect of the edge on the survival phenotype under pre-specified effecting edge settings, with the SNP effect obtained from the DPR model. Simulations were conducted with four different between-node correlation patterns (the combination of sine and quadratic, sine, quadratic, and linear) and three different censoring rates (0.1, 0.3, and 0.5). (**a**) Only an edge has an effect. (**b**) Both node and edge have effects, with the effecting node hanging on the edge. (**c**) Both node and edge have effects, with the effecting node not hanging on the edge.

Then, we also evaluated the ability of both methods in identifying the significant edge. Table 3 summarizes the estimated type I error for survival phenotype with a sample size of 5000 under three scenarios. Similarly, the type I error rates of CoNet and CPNT remain calibrated with different correlation patterns among nodes, the censoring rate, and the sample size (Tables S3 and S4). Figure 3 shows the power for survival phenotype when only an edge has a pre-specified effect. With the same settings as in the detecting node effect, we can draw the same conclusions as for the power of both methods. With nonlinear correlation between the nodes inside the network, the power of CoNet has a better performance than that of CPNT. For instance, under a combination of sine and quadratic relationships, with a sample size of 20,000 and censoring rate of 0.1, the power of CoNet is 0.449 and the power of CPNT is 0.042 (Figure 3a). Although the power of CoNet decreases to 0.298 when the censoring rate increases to 0.5, it is still higher than that of CPNT (0.043) (Figure 3a). 

As expected, the type of nonlinear pattern plays a key role in the difference of power for both methods. For example, the power of CoNet is much higher than that of CPNT when the relationship between nodes is a combination of both sine and quadratic, while they have a comparable performance when nodes are in the sine relationship. As the sample size increases and the censoring rate decreases, the power of CoNet increases even more dramatically. For example, if the nodes are correlated within a pattern of recombination of sine and quadratic, when the sample size increases from 5000 to 20,000 with a fixed censoring rate = 0.1, the power of CoNet increases from 0.142 to 0.449 whereas the power of CPNT increases from 0.038 to 0.042 (Figure 3a). If the nodes are correlated within a pattern of the combination of sine and quadratic, when the censoring rate decreases from 0.3 to 0.1 with a fixed sample size = 20,000, the power of CoNet increases from 0.376 to 0.449 while the power of CPNT increases from 0.037 to 0.042 (Figure 3a). 

The power advantage of CoNet over CPNT remains with nonlinear correlation patterns, regardless of the censoring rate. Together, these results illustrate that CoNet is more powerful to capture the nonlinear relationships than linear ones. Note that, CoNet only just has a slightly lower power than CPNT with the linear correlation pattern, possibly because the PM is the gold standard to capture the linear correlation in this case. We can obtain the similar findings when both the node and edge had effects, either with the effecting node hanging on the edge or with the effecting node not hanging on the edge.

Similar results can also be found when randomly selecting the effecting nodes and effecting edges (Tables S5–S10, Figures S1 and S2) and when the predicted value of gene expression was calculated by the BSLMM model (Tables S11–S22, Figures S3–S6). For example, in the setting that only an edge has a pre-specified effect, under a combination of sine and quadratic relationships with a sample size of 20,000 and censoring rate of 0.1, the power of CoNet and CPNT is 0.449 and 0.042 using the DPR model (Figure 3a) and 0.465 and 0.053 using the BSLMM model (Figure S4a).

Additional simulations also illustrated the advantage of CoNet with a high censoring rate (Tables S23 and S24, Figures S8 and S9) and the robustness of CoNet when several genes are unavailable (Table S26–S31, Figures S10 and S11). In addition, CoNet is computationally efficient (Table S25).

### 3.2. Application

We completely analyzed 7 networks, including 338 nodes and 440 edges. Overall, CoNet successfully identified 3 genes and 7 edges (Table 1 and Table 4), while CPNT identified 3 genes and failed to identify any edges. Consistent with the simulations that both methods have a comparable performance in detecting the node effect, both methods successfully identified 3 genes, respectively, including the common genes *CDK6* in hsa04115 (adjusted *p* = 0.048 for CoNet and 0.043 for CPNT) and *DTX3L* in hsa04330 (adjusted *p* = 0.034 for CoNet and 0.012 for CPNT). The significant node identified by CoNet rather than CPNT was *IL6* (adjusted *p* = 0.012 for CoNet and 0.156 for CPNT) in hsa04623. The significant node identified by CPNT rather than CoNet was *MAML2* in hsa04330 (adjusted *p* = 0.096 for CoNet and 0.043 for CPNT).

The significant edges identified only by CoNet included *GADD45A_CDK1* (adjusted *p* = 0.027) in hsa04115, *DDX58_TRIM25* (adjusted *p* = 1.15 × 10^−3^) in hsa04622, *MAP3K1_MAPK12* (adjusted *p* = 0.027) in hsa04622, *VHL_EP300* (adjusted *p* = 5.78 × 10^−6^) in hsa05211, *NEDD4L_SFN* (adjusted *p* = 3.28 × 10^−3^) in hsa04960, *NOTCH2_DVL2* (adjusted *p* = 0.011) in hsa04330, and *NOTCH4_DVL2* (*p* = 1.26 × 10^−3^, hsa04330). Two exemplary scatter plots (Figure S7a–d) further illustrated that the joint distribution of the two nodes linked by the significant edges may be different between the individuals with a short survival time (less than 25% quantile) and a long survival time (higher than the 75% quantile). 

## 4. Discussion

It is essential to identify biological networks that are associated with complex traits to understand the network mechanism related to complex diseases. In this study, we proposed CoNet, a novel statistical method for detecting the association between one given network and the survival time. CoNet applies DPR to find the gene expression prediction weights, then implements PMI to quantify the correlations between the nodes. Moreover, CoNet can provide the significant effecting gene nodes and edges associated with the survival outcomes at once. CoNet uses nonparametric kernel density estimation to calculate PMI between two genes. Here, we demonstrated several benefits of CoNet through extensive simulations and real data analysis. 

It could be argued that the PMI estimate could be obtained among the network nodes of observed gene expression from the eQTL study instead of predicted gene expression in GWAS. The standard TWAS analysis could then be performed using the Cox proportional hazards model in the second stage, using the PMI estimate as a new exposure. However, the eQTL study would have a large prediction error because of its limited sample size (e.g., only 465 samples in the GEUVADIS data). In addition, unlike traditional TWAS analysis that uses the cis-SNPs of each gene as the genotypes, it is challenging, both biologically and statistically, to determine the SNPs that are suitable for the PMI between two genes as the genotypes. 

In real data analysis, we found several genes or gene–gene interactions associated with breast cancer. As for the specific genes, *CDK6* and *DTX3L* were identified by both CoNet and CPNT, while *IL6* was identified by CoNet only. *CDK6* is a known classic cell cycle kinase that facilitates the progression of cells. Some studies have detected *CDK6* mRNA expression increases in breast cancer tissues versus that in adjacent tissues [33]. *DTX3L* is found to be overexpressed in breast cancer, which functions as a negative regulator of ATRA-induced growth inhibition in breast cancer cells [34]. *IL-6* was shown to promote or inhibit the growth of breast cancer cells. Indeed, some studies considered *IL-6* as a potential marker in the prognosis of breast cancer [35]. As for the significant edges, CoNet identified *GADD45A_CDK1*, *DDX58_TRIM25*, *MAP3K1_MAPK12*, *VHL_EP300*, *NEDD4L_SFN*, *NOTCH2_DVL2*, and *NOTCH4_DVL2*, but CPNT failed to identify any edges. Previous studies confirmed that *SFN* inhibited TGF-β1-induced migration and invasion in breast cancer cells [36] and *NEDD4L* expression significantly reduced in breast invasive carcinoma [37]. In $ER^{+}$- tumor tissues, the mRNA levels of *DDX58* were significantly higher than in adjacent tissues [38]. Similarly, *TRIM25* was reported as overexpressed in breast cancer cells [39]. 

CoNet is not without limitations. Firstly, only CPNT was used as a reference to evaluate the performance of CoNet. We have performed additional simulations to compare CoNet with the modified TIGAR, where we changed the linear regression in the second stage of TIGAR to be the Cox model. The results illustrated that CoNet has a higher power than TIGAR (Table S32, Figure S12). Secondly, it is assumed that the network structure is prior known. The learning network structure needs to identify all the possible edges that match the data. Often, a joint probability distribution of an all-gene network can reveal multiple network structures. In CoNet, the PMI estimation for different gene expression predictions is directly plugged into the regression model, ignoring the accuracy of the PMI estimator, particularly in eQTL studies with a small sample size. The interpretation of the regression coefficients of the edges (PMI) is correlation pattern specific. Intuitively, the positive coefficient indicates that the hazard will increase as the strength of the non-independency between the two node variables increases. The negative effect indicates that the hazard will decrease as the strength of the non-independency between the two node variables increases. Even so, the effect size should be interpreted in caution. In addition, the interaction studies in this research are statistical interactions. Indeed, it is hard to define what the specific biological interactions are. They can be proteins coded by two interaction genes working in the same pathway, wherein one gene affects the expression of the other, or both genes sharing a common regulatory mechanism. However, despite these limitations, our study highlights that CoNet is an appealing approach for simultaneously identifying the potential nodes and edges that are related to the survival time in large datasets.

## 5. Conclusions

The proposed method here, CoNet, effectively accounts for network structure and can capture and quantify the general relationship among different genes. It is robust against different model assumptions of genetic effect sizes and different censoring rates and can simultaneously identify the potential nodes and edges that are related to the survival time in TWAS.

## Figures and Tables

**Table 1:** Summary of analyzed networks.

Network	KEGG		Overlap	
	Node	Edge	Node	Edge
hsa04115	73	88	68	82
hsa04330	59	164	57	131
hsa04623	75	100	60	73
hsa04960	37	37	31	31
hsa04622	71	147	53	86
hsa05211	68	98	62	31
hsa04630-nt06219	7	6	7	6
Total	390	640	338	440

**Table 2:** The type I error for detecting the effect of the node on the survival phenotype under three scenarios where the effecting node is pre-specified (*n* = 5000), with the SNP effect obtained from DPR model. Simulations were conducted with four different between-node correlation patterns (the combination of sine and quadratic, sine, quadratic, and linear) and three different censoring rates (0.1, 0.3, and 0.5).

Scenario 1: Only Node Changes				
Correlation patterns	Methods	Censoring rate		
		0.1	0.3	0.5
$x_{k}=0.5x_{l}$	CoNet	0.051	0.050	0.046
	CPNT	0.051	0.047	0.048
$x_{k}=0.1x_{l}{}^{2}$	CoNet	0.038	0.049	0.043
	CPNT	0.039	0.050	0.044
$x_{k}=sinx_{l}$	CoNet	0.037	0.047	0.043
	CPNT	0.040	0.048	0.045
$x_{k}=sin^{2}x_{l}$	CoNet	0.037	0.045	0.043
	CPNT	0.040	0.050	0.045
Scenario 2: Both node and edge change with node hanging on the edge				
Correlation patterns	Methods	Censoring rate		
		0.1	0.3	0.5
$x_{k}=0.5x_{l}$	CoNet	0.044	0.046	0.047
	CPNT	0.048	0.044	0.051
$x_{k}=0.1x_{l}{}^{2}$	CoNet	0.051	0.047	0.055
	CPNT	0.057	0.049	0.052
$x_{k}=sinx_{l}$	CoNet	0.046	0.048	0.054
	CPNT	0.046	0.046	0.054
$x_{k}=sin^{2}x_{l}$	CoNet	0.058	0.055	0.055
	CPNT	0.053	0.045	0.053
Scenario 3: Both node and edge change with node not hanging on the edge				
Correlation patterns	Methods	Censoring rate		
		0.1	0.3	0.5
$x_{k}=0.5x_{l}$	CoNet	0.047	0.061	0.068
	CPNT	0.044	0.055	0.064
$x_{k}=0.1x_{l}{}^{2}$	CoNet	0.069	0.068	0.061
	CPNT	0.060	0.071	0.054
$x_{k}=sinx_{l}$	CoNet	0.069	0.069	0.061
	CPNT	0.058	0.071	0.057
$x_{k}=sin^{2}x_{l}$	CoNet	0.068	0.068	0.062
	CPNT	0.059	0.072	0.058

**Table 3:** The type I error for testing the effect of the edge on the survival phenotype under three scenarios where the effecting edge is pre-specified (*n* = 5000), with the SNP effect obtained from DPR model. Simulations were conducted with four different between-node correlation patterns (the combination of sine and quadratic, sine, quadratic, and linear) and three different censoring rates (0.1, 0.3, and 0.5).

Scenario 1: Only Edge Changes				
Correlation patterns	Methods	Censoring rates		
		0.1	0.3	0.5
$x_{k}=0.5x_{l}$	CoNet	0.049	0.039	0.043
	CPNT	0.046	0.040	0.043
$x_{k}=0.1x_{l}{}^{2}$	CoNet	0.055	0.058	0.054
	CPNT	0.042	0.055	0.056
$x_{k}=sinx_{l}$	CoNet	0.040	0.048	0.049
	CPNT	0.040	0.054	0.052
$x_{k}=sin^{2}x_{l}$	CoNet	0.050	0.050	0.042
	CPNT	0.043	0.058	0.060
Scenario 2: Both node and edge change with node hanging on the edge				
Correlation patterns	Methods	Censoring rates		
		0.1	0.3	0.5
$x_{k}=0.5x_{l}$	CoNet	0.051	0.053	0.049
	CPNT	0.053	0.060	0.051
$x_{k}=0.1x_{l}{}^{2}$	CoNet	0.056	0.052	0.054
	CPNT	0.056	0.053	0.046
$x_{k}=sinx_{l}$	CoNet	0.058	0.060	0.053
	CPNT	0.056	0.058	0.058
$x_{k}=sin^{2}x_{l}$	CoNet	0.042	0.044	0.039
	CPNT	0.057	0.056	0.050
Scenario 3: Both node and edge change with node not hanging on the edge				
Correlation patterns	Methods	Censoring rates		
		0.1	0.3	0.5
$x_{k}=0.5x_{l}$	CoNet	0.069	0.044	0.063
	CPNT	0.059	0.039	0.055
$x_{k}=0.1x_{l}{}^{2}$	CoNet	0.056	0.052	0.055
	CPNT	0.056	0.053	0.052
$x_{k}=sinx_{l}$	CoNet	0.064	0.057	0.053
	CPNT	0.059	0.056	0.058
$x_{k}=sin^{2}x_{l}$	CoNet	0.043	0.046	0.039
	CPNT	0.066	0.054	0.050

**Table 4:** Significantly affecting nodes and edge for two methods with *p*-values being corrected by FDR.

	CoNet		CPNT	
	Node	Edge	Node	Edge
hsa04115	*CDK6* (*p* = 0.048)	*GADD45A_CDK1* (*p* = 0.027)	*CDK6* (*p* = 0.043)	/
hsa04623	*IL6* (*p* = 0.012)	/	/	/
hsa04622	/	*DDX58_TRIM25* (*p* = 1.15 × 10^−3^) *MAP3K1_MAPK12* (*p* = 0.027)	/	/
hsa05211	/	*VHL_EP300* (*p* = 5.78 × 10^−6^)	/	/
hsa04960		*NEDD4L_SFN* (*p* = 3.28 × 10^−3^)	/	
hsa04330	*DTX3L* (*p* = 0.034) *MAML2* (*p* = 0.096)	*NOTCH2_DVL2* (*p* = 0.011) *NOTCH4_DVL2*(*p* = 1.26 × 10^−3^)	*DTX3L* (*p* = 0.012) *MAML2*(*p* = 0.043)	/

## Data Availability

The GEUVADIS data underlying this article are available at http://www.geuvadis.org (accessed on 20 April 2022). The breast cancer data of the UK Biobank are available at https://www.ukbiobank.ac.uk/ (accessed on 15 June 2022), with application number 51470. The sample QC procedure in Neale lab is available at https://github.com/Nealelab/UK_Biobank_GWAS/tree/master/imputed-v2-gwas (accessed on 20 April 2022).

## Supplementary Materials

The following supporting information can be downloaded at: https://www.mdpi.com/article/10.3390/genes14030586/s1, Figure S1: Power for the effecting node is randomly selected, using DPR as imputation model; Figure S2: Power for the effecting edge is randomly selected, using DPR as imputation model; Figure S3: Power for the effecting node is pre-specified, using BSLMM as imputation model; Figure S4: Power for the effecting edge is pre-specified, using BSLMM as imputation model; Figure S5: Power for the effecting node is randomly selected, using BSLMM as imputation model; Figure S6: Power for the effecting edge is randomly selected, using BSLMM as imputation model; Figure S7: Scatter plot of the expression of two different genes with different survival status; Figure S8: Power for the effecting node and the effecting edge are pre-specified, using DPR as imputation model (including high censoring rate of 0.7); Figure S9: Power for the effecting node and the effecting edge are randomly selected, using DPR as imputation model (including high censoring rate of 0.7); Figure S10: Power of CoNet in a reduced gene network with some genes being unavailable, where the effecting node and effecting edge are pre-specified; Figure S11: Power of CoNet in a reduced gene network with some genes being unavailable, where the effecting node and effecting edge are randomly selected; Figure S12: Power of CoNet and TIGAR to test the effect of nodes under the effect size of between-node correlation being 0.1; Table S1: Type I error for the effecting node is pre-specified, using DPR as imputation model (*n* = 10,000); Table S2: Type I error for the effecting node is pre-specified, using DPR as imputation model (*n* = 20,000); Table S3: Type I error for the effecting edge is pre-specified, using DPR as imputation model (*n* = 10,000); Table S4: Type I error for the effecting edge is pre-specified, using DPR as imputation model (*n* = 20,000); Table S5: Type I error for the effecting node is randomly selected, using DPR as imputation model (*n* = 5000); Table S6: Type I error for the effecting node is randomly selected, using DPR as imputation model (*n* = 10,000); Table S7: Type I error for the effecting node is randomly selected, using DPR as imputation model (*n* = 20,000); Table S8: Type I error for the effecting edge is randomly selected, using DPR as imputation model (*n* = 5000); Table S9: Type I error for the effecting edge is randomly selected, using DPR as imputation model (*n* = 10,000); Table S10: Type I error for the effecting edge is randomly selected, using DPR as imputation model (*n* = 20,000); Table S11: Type I error for the effecting node is pre-specified, using BSLMM as imputation model (*n* = 5000); Table S12: Type I error for the effecting node is pre-specified, using BSLMM as imputation model (*n* = 10,000); Table S13: Type I error for the effecting node is pre-specified, using BSLMM as imputation model (*n* = 20,000); Table S14: Type I error for the effecting edge is pre-specified, using BSLMM as imputation model (*n* = 5000); Table S15: Type I error for the effecting edge is pre-specified, using BSLMM as imputation model (*n* = 10,000); Table S16: Type I error for the effecting edge is pre-specified, using BSLMM as imputation model (*n* = 20,000); Table S17: Type I error for the effecting node is randomly selected, using BSLMM as imputation model (*n* = 5000); Table S18: Type I error for the effecting node is randomly selected, using BSLMM as imputation model (*n* = 10,000); Table S19: Type I error for the effecting node is randomly selected, using BSLMM as imputation model (*n* = 20,000); Table S20: Type I error for the effecting edge is randomly selected, using BSLMM as imputation model (*n* = 5000); Table S21: Type I error for the effecting edge is randomly selected, using BSLMM as imputation model (*n* = 10,000); Table S22: Type I error for the effecting edge is randomly selected, using BSLMM as imputation model (*n* = 20,000); Table S23: Type I error for the effecting node and the effecting edge are pre-specified, using DPR as imputation model(censoring rate = 0.7); Table S24: Type I error for the effecting node and the effecting edge are randomly selected, using DPR as imputation model(censoring rate = 0.7); Table S25: Mean computational time (seconds) of both methods; Table S26: Type I error of CoNet in a reduced gene network with some proportions (0, 20%, 30%) of genes being unavailable, where the effecting node and effecting edge are pre-specified (*n* = 5000); Table S27: Type I error of CoNet in a reduced gene network with some proportions (0, 20%, 30%) of genes being unavailable, where the effecting node and effecting edge are pre-specified (*n* = 10,000); Table S28: Type I error of CoNet in a reduced gene network with some proportions (0, 20%, 30%) of genes being unavailable, where the effecting node and effecting edge are pre-specified (*n* = 20,000); Table S29: Type I error of CoNet in a reduced gene network with some proportions (0, 20%, 30%) of genes being unavailable, where the effecting node and effecting edge are randomly selected (*n* = 5000); Table S30: Type I error of CoNet in a reduced gene network with some proportions (0, 20%, 30%) of genes being unavailable, where the effecting node and effecting edge are randomly selected (*n* = 10,000); Table S31: Type I error of CoNet in a reduced gene network with some proportions (0, 20%, 30%) of genes being unavailable, where the effecting node and effecting edge are randomly selected (*n* = 20,000); Table S32: Type I error of CoNet and TIGAR to test the effect of nodes under the survival phenotype.
