# Supplementary material for: CoNet: Efficient Network Regression for Survival Analysis in Transcriptome-Wide Association Studies—With Applications to Studies of Breast Cancer
Source: Genes (Basel). 2023 Feb 25;14(3):586. doi: 10.3390/genes14030586 (PMC10048118; doi:10.3390/genes14030586)
Supplement: Supplementary file 1 [file genes-14-00586-s001.zip › genes-2150425-supplementary.pdf]

Table S1. The type I error for detecting the effect of the node on the survival phenotype under three scenarios where the effecting node is pre-specified (n=10000), with DPR as the gene expression prediction model in TWAS. Simulations were conducted with four different between-node correlation patterns (the recombination of quadratic and sine, sine, quadratic, and linear) and three different censoring rates (0.1, 0.3, and 0.5).

**Scenario 1 :Only node changes**				
Correlation patterns	Methods	Censoring rate		
		0.1	0.3	0.5
$x_k = 0.5x_l$	CoNet	0.047	0.052	0.057
	CPNT	0.051	0.054	0.053
$x_k = 0.1{x_l}^2$	CoNet	0.045	0.050	0.062
	CPNT	0.048	0.052	0.057
$x_k = sinx_l$	CoNet	0.043	0.050	0.059
	CPNT	0.048	0.053	0.058
$x_k = sin^2x_l$	CoNet	0.042	0.050	0.061
	CPNT	0.047	0.052	0.057
**Scenario 2 : Both node and edge change with node hanging on edge**				
Correlation patterns	Methods	Censoring rate		
		0.1	0.3	0.5
$x_k = 0.5x_l$	CoNet	0.045	0.043	0.045
	CPNT	0.045	0.047	0.041
$x_k = 0.1{x_l}^2$	CoNet	0.055	0.057	0.047
	CPNT	0.050	0.051	0.046
$x_k = sinx_l$	CoNet	0.036	0.051	0.046
	CPNT	0.036	0.046	0.045
$x_k = sin^2x_l$	CoNet	0.044	0.051	0.049
	CPNT	0.046	0.054	0.052
**Scenario 3: Both node and edge change with node not hanging on edge**				
Correlation patterns	Methods	Censoring rate		
		0.1	0.3	0.5
$x_k = 0.5x_l$	CoNet	0.048	0.048	0.046
	CPNT	0.054	0.052	0.045
$x_k = 0.1{x_l}^2$	CoNet	0.055	0.054	0.059
	CPNT	0.054	0.050	0.055
$x_k = sinx_l$	CoNet	0.054	0.053	0.057
	CPNT	0.055	0.048	0.053
$x_k = sin^2x_l$	CoNet	0.054	0.053	0.057
	CPNT	0.056	0.050	0.054

Table S2. The type I error for detecting the effect of the node on the survival phenotype under three scenarios where the effecting node is pre-specified (n=20000), with DPR as the gene expression prediction model in TWAS. Simulations were conducted with four different between-node correlation patterns (the recombination of quadratic and sine, sine, quadratic, and linear) and three different censoring rates (0.1, 0.3, and 0.5).

**Scenario 1 :Only node changes**				
Correlation patterns	Methods	Censoring rate		
		0.1	0.3	0.5
$x_k = 0.5x_l$	CoNet	0.051	0.055	0.048
	CPNT	0.048	0.055	0.043
$x_k = 0.1{x_l}^2$	CoNet	0.056	0.059	0.052
	CPNT	0.043	0.056	0.054
$x_k = sinx_l$	CoNet	0.057	0.057	0.051
	CPNT	0.054	0.055	0.055
$x_k = sin^2x_l$	CoNet	0.055	0.057	0.053
	CPNT	0.054	0.056	0.054
**Scenario 2 : Both node and edge change with node hanging on edge**				
Correlation patterns	Methods	Censoring rate		
		0.1	0.3	0.5
$x_k = 0.5x_l$	CoNet	0.049	0.046	0.057
	CPNT	0.043	0.048	0.057
$x_k = 0.1{x_l}^2$	CoNet	0.051	0.049	0.051
	CPNT	0.055	0.057	0.050
$x_k = sinx_l$	CoNet	0.041	0.056	0.042
	CPNT	0.047	0.058	0.048
$x_k = sin^2x_l$	CoNet	0.041	0.053	0.056
	CPNT	0.039	0.054	0.052
**Scenario 3: Both node and edge change with node not hanging on edge**				
Correlation patterns	Methods	Censoring rate		
		0.1	0.3	0.5
$x_k = 0.5x_l$	CoNet	0.054	0.060	0.051
	CPNT	0.062	0.059	0.050
$x_k = 0.1{x_l}^2$	CoNet	0.065	0.055	0.061
	CPNT	0.065	0.054	0.061
$x_k = sinx_l$	CoNet	0.050	0.053	0.055
	CPNT	0.052	0.052	0.053
$x_k = sin^2x_l$	CoNet	0.049	0.053	0.053
	CPNT	0.051	0.054	0.054

Table S3. The type I error for detecting the effect of the edge on the survival phenotype under three scenarios where the effecting edge is pre-specified (n=10000), with DPR as the gene expression prediction model in TWAS. Simulations were conducted with four different between-node correlation patterns (the recombination of quadratic and sine, sine, quadratic, and linear) and three different censoring rates (0.1, 0.3, and 0.5).

Correlation patterns	Methods	Censoring rate		
		0.1	0.3	0.5
**Scenario 1 :Only edge changes**				
$x_k = 0.5x_l$	CoNet	0.040	0.040	0.030
	CPNT	0.047	0.046	0.048
$x_k = 0.1{x_l}^2$	CoNet	0.059	0.049	0.046
	CPNT	0.068	0.061	0.061
$x_k = sinx_l$	CoNet	0.035	0.052	0.052
	CPNT	0.054	0.055	0.048
$x_k = sin^2x_l$	CoNet	0.062	0.058	0.069
	CPNT	0.054	0.049	0.053
**Scenario 2 : Both node and edge change with node hanging on edge**				
$x_k = 0.5x_l$	CoNet	0.063	0.060	0.065
	CPNT	0.056	0.062	0.049
$x_k = 0.1{x_l}^2$	CoNet	0.043	0.047	0.047
	CPNT	0.052	0.049	0.035
$x_k = sinx_l$	CoNet	0.040	0.046	0.055
	CPNT	0.050	0.045	0.060
$x_k = sin^2x_l$	CoNet	0.042	0.040	0.041
	CPNT	0.043	0.033	0.054
**Scenario 3: Both node and edge change with node not hanging on edge**				
$x_k = 0.5x_l$	CoNet	0.054	0.065	0.059
	CPNT	0.052	0.055	0.059
$x_k = 0.1{x_l}^2$	CoNet	0.043	0.047	0.047
	CPNT	0.052	0.055	0.048
$x_k = sinx_l$	CoNet	0.041	0.048	0.055
	CPNT	0.049	0.056	0.060
$x_k = sin^2x_l$	CoNet	0.043	0.038	0.041
	CPNT	0.048	0.051	0.054

Table S4. The type I error for detecting the effect of the edge on the survival phenotype under three scenarios where the effecting edge is pre-specified (n=20000), with DPR as the gene expression prediction model in TWAS. Simulations were conducted with four different between-node correlation patterns (the recombination of quadratic and sine, sine, quadratic, and linear) and three different censoring rates (0.1, 0.3, and 0.5).

Correlation patterns	Methods	Censoring rate		
		0.1	0.3	0.5
**Scenario 1 :Only edge changes**				
$x_k = 0.5x_l$	CoNet	0.052	0.053	0.060
	CPNT	0.062	0.060	0.064
$x_k = 0.1{x_l}^2$	CoNet	0.037	0.049	0.048
	CPNT	0.058	0.051	0.043
$x_k = sinx_l$	CoNet	0.044	0.054	0.049
	CPNT	0.047	0.053	0.039
$x_k = sin^2x_l$	CoNet	0.046	0.049	0.045
	CPNT	0.056	0.049	0.043
**Scenario 2 : Both node and edge change with node hanging on edge**				
Correlation patterns	Methods	Censoring rate		
		0.1	0.3	0.5
$x_k = 0.5x_l$	CoNet	0.059	0.045	0.046
	CPNT	0.053	0.056	0.050
$x_k = 0.1{x_l}^2$	CoNet	0.050	0.054	0.057
	CPNT	0.054	0.059	0.062
$x_k = sinx_l$	CoNet	0.039	0.047	0.054
	CPNT	0.040	0.055	0.059
$x_k = sin^2x_l$	CoNet	0.054	0.054	0.045
	CPNT	0.044	0.042	0.050
**Scenario 3: Both node and edge change with node not hanging on edge**				
Correlation patterns	Methods	Censoring rate		
		0.1	0.3	0.5
$x_k = 0.5x_l$	CoNet	0.057	0.053	0.056
	CPNT	0.063	0.056	0.053
$x_k = 0.1{x_l}^2$	CoNet	0.043	0.045	0.047
	CPNT	0.056	0.046	0.053
$x_k = sinx_l$	CoNet	0.049	0.047	0.054
	CPNT	0.052	0.046	0.059
$x_k = sin^2x_l$	CoNet	0.057	0.052	0.045
	CPNT	0.047	0.041	0.050

Table S5. The type I error for detecting the effect of the node on the survival phenotype under three scenarios where the effecting node is randomly selected (n=5000), with DPR as the gene expression prediction model in TWAS. Simulations were conducted with four different between-node correlation patterns (the recombination of quadratic and sine, sine, quadratic, and linear) and three different censoring rates (0.1, 0.3, and 0.5).

Correlation patterns	Methods	Censoring rate		
		0.1	0.3	0.5
**Scenario 1 :Only node changes**				
$x_k = 0.5x_l$	CoNet	0.049	0.054	0.047
	CPNT	0.047	0.056	0.056
$x_k = 0.1{x_l}^2$	CoNet	0.049	0.054	0.050
	CPNT	0.052	0.053	0.054
$x_k = sinx_l$	CoNet	0.047	0.055	0.048
	CPNT	0.051	0.056	0.053
$x_k = sin^2x_l$	CoNet	0.046	0.052	0.048
	CPNT	0.052	0.053	0.054
**Scenario 2 : Both node and edge change with node hanging on edge**				
Correlation patterns	Methods	Censoring rate		
		0.1	0.3	0.5
$x_k = 0.5x_l$	CoNet	0.041	0.047	0.043
	CPNT	0.040	0.048	0.030
$x_k = 0.1{x_l}^2$	CoNet	0.058	0.060	0.058
	CPNT	0.057	0.066	0.055
$x_k = sinx_l$	CoNet	0.049	0.060	0.053
	CPNT	0.048	0.067	0.057
$x_k = sin^2x_l$	CoNet	0.048	0.060	0.057
	CPNT	0.049	0.066	0.057
**Scenario 3: Both node and edge change with node not hanging on edge**				
Correlation patterns	Methods	Censoring rate		
		0.1	0.3	0.5
$x_k = 0.5x_l$	CoNet	0.065	0.058	0.046
	CPNT	0.064	0.059	0.049
$x_k = 0.1{x_l}^2$	CoNet	0.038	0.048	0.044
	CPNT	0.032	0.036	0.043
$x_k = sinx_l$	CoNet	0.036	0.043	0.046
	CPNT	0.043	0.040	0.041
$x_k = sin^2x_l$	CoNet	0.036	0.045	0.045
	CPNT	0.039	0.035	0.043

Table S6. The type I error for detecting the effect of the node on the survival phenotype under three scenarios where the effecting node is randomly selected (n=10000), with DPR as the gene expression prediction model in TWAS. Simulations were conducted with four different between-node correlation patterns (the recombination of quadratic and sine, sine, quadratic, and linear) and three different censoring rates (0.1, 0.3, and 0.5).

Correlation patterns	Methods	Censoring rate 0.1	Censoring rate 0.3	Censoring rate 0.5
**Scenario 1 :Only node changes**				
$x_k = 0.5x_l$	CoNet	0.036	0.047	0.054
	CPNT	0.035	0.051	0.064
$x_k = 0.1{x_l}^2$	CoNet	0.031	0.042	0.040
	CPNT	0.037	0.045	0.041
$x_k = sinx_l$	CoNet	0.070	0.060	0.047
	CPNT	0.063	0.068	0.052
$x_k = sin^2x_l$	CoNet	0.062	0.044	0.035
	CPNT	0.063	0.045	0.040
**Scenario 2 : Both node and edge change with node hanging on edge**				
$x_k = 0.5x_l$	CoNet	0.049	0.050	0.045
	CPNT	0.043	0.049	0.048
$x_k = 0.1{x_l}^2$	CoNet	0.055	0.054	0.046
	CPNT	0.056	0.058	0.045
$x_k = sinx_l$	CoNet	0.050	0.057	0.047
	CPNT	0.049	0.052	0.045
$x_k = sin^2x_l$	CoNet	0.049	0.056	0.051
	CPNT	0.049	0.056	0.045
**Scenario 3: Both node and edge change with node not hanging on edge**				
$x_k = 0.5x_l$	CoNet	0.036	0.048	0.050
	CPNT	0.037	0.049	0.054
$x_k = 0.1{x_l}^2$	CoNet	0.049	0.055	0.050
	CPNT	0.050	0.050	0.046
$x_k = sinx_l$	CoNet	0.034	0.047	0.054
	CPNT	0.031	0.040	0.050
$x_k = sin^2x_l$	CoNet	0.037	0.044	0.056
	CPNT	0.032	0.043	0.056

Table S7. The type I error for detecting the effect of the node on the survival phenotype under three scenarios where the effecting node is randomly selected (n=20000), with DPR as the gene expression prediction model in TWAS. Simulations were conducted with four different between-node correlation patterns (the recombination of quadratic and sine, sine, quadratic, and linear) and three different censoring rates (0.1, 0.3, and 0.5).

**Scenario 1 :Only node changes**				
Correlation patterns	Methods	Censoring rate		
		0.1	0.3	0.5
$x_k = 0.5x_l$	CoNet	0.056	0.056	0.053
	CPNT	0.055	0.052	0.053
$x_k = 0.1{x_l}^2$	CoNet	0.070	0.064	0.051
	CPNT	0.064	0.068	0.050
$x_k = sinx_l$	CoNet	0.070	0.060	0.047
	CPNT	0.063	0.068	0.052
$x_k = sin^2x_l$	CoNet	0.047	0.061	0.048
	CPNT	0.049	0.068	0.050
**Scenario 2 : Both node and edge change with node hanging on edge**				
Correlation patterns	Methods	Censoring rate		
		0.1	0.3	0.5
$x_k = 0.5x_l$	CoNet	0.053	0.052	0.058
	CPNT	0.054	0.053	0.064
$x_k = 0.1{x_l}^2$	CoNet	0.049	0.051	0.051
	CPNT	0.055	0.051	0.049
$x_k = sinx_l$	CoNet	0.048	0.049	0.053
	CPNT	0.051	0.048	0.049
$x_k = sin^2x_l$	CoNet	0.048	0.049	0.052
	CPNT	0.051	0.050	0.049
**Scenario 3: Both node and edge change with node not hanging on edge**				
Correlation patterns	Methods	Censoring rate		
		0.1	0.3	0.5
$x_k = 0.5x_l$	CoNet	0.042	0.050	0.046
	CPNT	0.044	0.050	0.046
$x_k = 0.1{x_l}^2$	CoNet	0.060	0.051	0.042
	CPNT	0.060	0.053	0.042
$x_k = sinx_l$	CoNet	0.050	0.055	0.061
	CPNT	0.052	0.053	0.060
$x_k = sin^2x_l$	CoNet	0.051	0.055	0.061
	CPNT	0.051	0.058	0.061

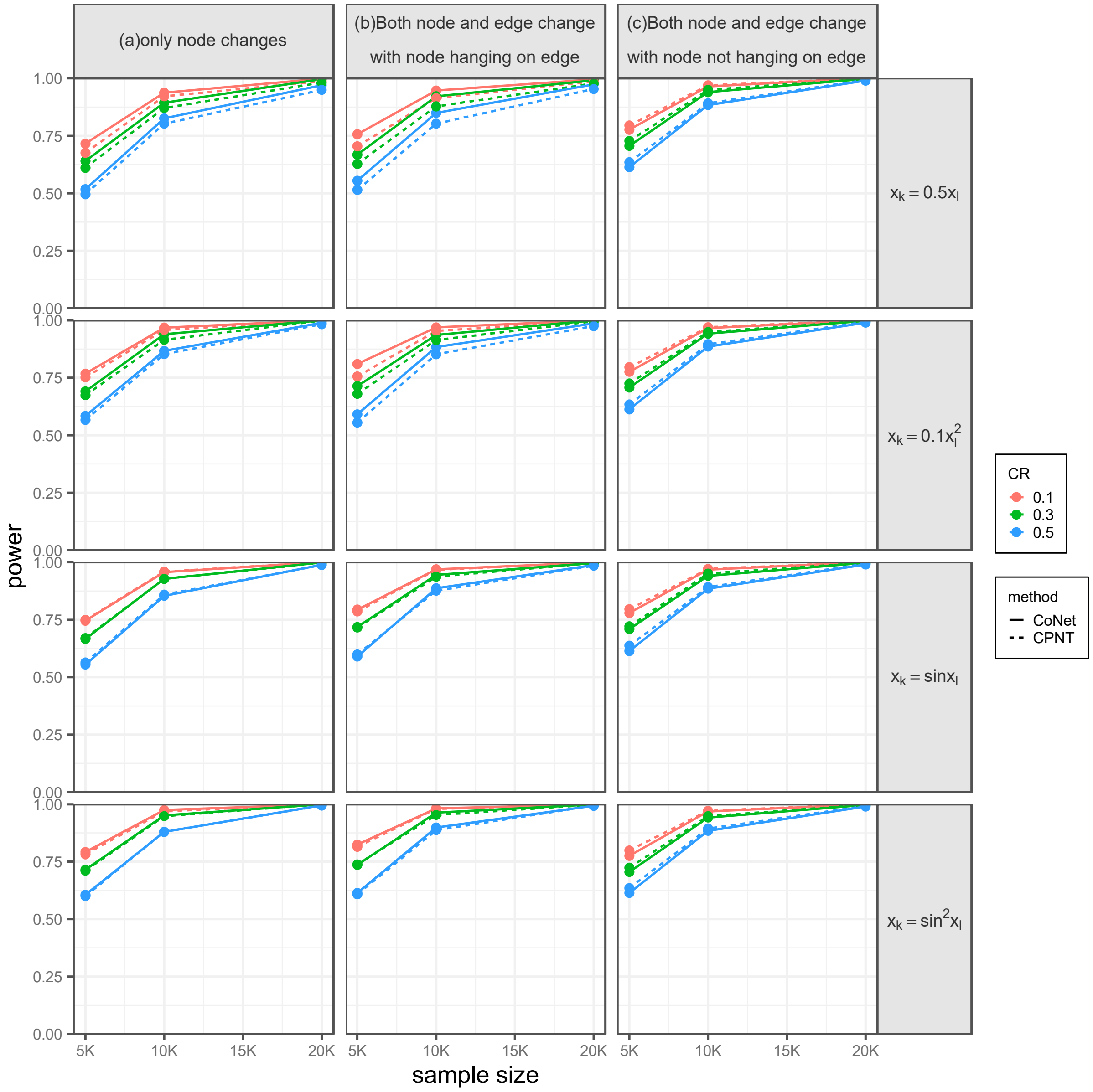

Figure S1. The power for detecting the effect of the node on the survival phenotype under the setting that the effecting node is randomly selected, with DPR as the gene expression prediction model in TWAS. Simulations were conducted with four different between-node correlation patterns (the recombination of quadratic and sine, sine, quadratic, and linear) and three different censoring rates (0.1, 0.3, and 0.5): (**a**) Only a node has an effect; (**b**) Both node and edge have effects, with the effecting node hanging on the edge; (**c**) Both node and edge have effects, with the effecting node not hanging on the edge.

Table S8. The type I error for detecting the effect of the edge on the survival phenotype under three scenarios where the effecting edge is randomly selected (n=5000), with DPR as the gene expression prediction model in TWAS. Simulations were conducted with four different between-node correlation patterns (the recombination of quadratic and sine, sine, quadratic, and linear) and three different censoring rates (0.1, 0.3, and 0.5).

**Scenario 1 :Only edge changes**				
Correlation patterns	Methods	Censoring rate		
		0.1	0.3	0.5
$x_k = 0.5x_l$	CoNet	0.041	0.043	0.053
	CPNT	0.048	0.045	0.0.50
$x_k = 0.1{x_l}^2$	CoNet	0.033	0.056	0.051
	CPNT	0.054	0.049	0.049
$x_k = sinx_l$	CoNet	0.041	0.049	0.054
	CPNT	0.052	0.050	0.045
$x_k = sin^2x_l$	CoNet	0.039	0.051	0.048
	CPNT	0.047	0.043	0.045
**Scenario 2 : Both node and edge change with node hanging on edge**				
Correlation patterns	Methods	Censoring rate		
		0.1	0.3	0.5
$x_k = 0.5x_l$	CoNet	0.042	0.048	0.050
	CPNT	0.035	0.041	0.058
$x_k = 0.1{x_l}^2$	CoNet	0.045	0.052	0.063
	CPNT	0.047	0.050	0.043
$x_k = sinx_l$	CoNet	0.040	0.053	0.059
	CPNT	0.046	0.048	0.046
$x_k = sin^2x_l$	CoNet	0.045	0.050	0.054
	CPNT	0.048	0.046	0.045
**Scenario 3: Both node and edge change with node not hanging on edge**				
Correlation patterns	Methods	Censoring rate		
		0.1	0.3	0.5
$x_k = 0.5x_l$	CoNet	0.046	0.048	0.059
	CPNT	0.047	0.056	0.058
$x_k = 0.1{x_l}^2$	CoNet	0.053	0.051	0.053
	CPNT	0.045	0.046	0.043
$x_k = sinx_l$	CoNet	0.040	0.047	0.049
	CPNT	0.043	0.048	0.047
$x_k = sin^2x_l$	CoNet	0.042	0.052	0.049
	CPNT	0.042	0.044	0.044

Table S9. The type I error for detecting the effect of the edge on the survival phenotype under three scenarios where the effecting edge is randomly selected (n=10000), with DPR as the gene expression prediction model in TWAS. Simulations were conducted with four different between-node correlation patterns (the recombination of quadratic and sine, sine, quadratic, and linear) and three different censoring rates (0.1, 0.3, and 0.5).

Scenario 1 :Only edge changes

Correlation patterns	Methods	Censoring rate		
		0.1	0.3	0.5
$x_k = 0.5x_l$	CoNet	0.047	0.058	0.068
	CPNT	0.036	0.043	0.043
$x_k = 0.1{x_l}^2$	CoNet	0.050	0.047	0.051
	CPNT	0.049	0.042	0.052
$x_k = sinx_l$	CoNet	0.035	0.046	0.058
	CPNT	0.044	0.045	0.052
$x_k = sin^2x_l$	CoNet	0.028	0.045	0.047
	CPNT	0.049	0.044	0.054

Scenario 2 : Both node and edge change with node hanging on edge

Correlation patterns	Methods	Censoring rate		
		0.1	0.3	0.5
$x_k = 0.5x_l$	CoNet	0.051	0.061	0.065
	CPNT	0.050	0.054	0.037
$x_k = 0.1{x_l}^2$	CoNet	0.058	0.051	0.053
	CPNT	0.042	0.045	0.049
$x_k = sinx_l$	CoNet	0.047	0.050	0.049
	CPNT	0.036	0.047	0.048
$x_k = sin^2x_l$	CoNet	0.051	0.052	0.048
	CPNT	0.038	0.045	0.050

Scenario 3: Both node and edge change with node not hanging on edge

Correlation patterns	Methods	Censoring rate		
		0.1	0.3	0.5
$x_k = 0.5x_l$	CoNet	0.033	0.033	0.033
	CPNT	0.046	0.050	0.051
$x_k = 0.1{x_l}^2$	CoNet	0.047	0.057	0.055
	CPNT	0.045	0.046	0.046
$x_k = sinx_l$	CoNet	0.045	0.051	0.057
	CPNT	0.052	0.060	0.060
$x_k = sin^2x_l$	CoNet	0.039	0.049	0.056
	CPNT	0.054	0.066	0.056

Table S10. The type I error for detecting the effect of the edge on the survival phenotype under three scenarios where the effecting edge is randomly selected (n=20000), with DPR as the gene expression prediction model in TWAS. Simulations were conducted with four different between-node correlation patterns (the recombination of quadratic and sine, sine, quadratic, and linear) and three different censoring rates (0.1, 0.3, and 0.5).

**Scenario 1 :Only edge changes**				
Correlation patterns	Methods	Censoring rate		
		0.1	0.3	0.5
$x_k = 0.5x_l$	CoNet	0.035	0.042	0.040
	CPNT	0.029	0.035	0.038
$x_k = 0.1{x_l}^2$	CoNet	0.052	0.044	0.047
	CPNT	0.041	0.041	0.044
$x_k = sinx_l$	CoNet	0.052	0.049	0.056
	CPNT	0.041	0.040	0.044
$x_k = sin^2x_l$	CoNet	0.049	0.052	0.050
	CPNT	0.039	0.043	0.043
**Scenario 2 : Both node and edge change with node hanging on edge**				
Correlation patterns	Methods	Censoring rate		
		0.1	0.3	0.5
$x_k = 0.5x_l$	CoNet	0.044	0.042	0.046
	CPNT	0.049	0.053	0.058
$x_k = 0.1{x_l}^2$	CoNet	0.062	0.063	0.054
	CPNT	0.066	0.057	0.048
$x_k = sinx_l$	CoNet	0.058	0.060	0.054
	CPNT	0.047	0.051	0.044
$x_k = sin^2x_l$	CoNet	0.055	0.050	0.054
	CPNT	0.050	0.056	0.047
**Scenario 3: Both node and edge change with node not hanging on edge**				
Correlation patterns	Methods	Censoring rate		
		0.1	0.3	0.5
$x_k = 0.5x_l$	CoNet	0.046	0.048	0.059
	CPNT	0.047	0.056	0.058
$x_k = 0.1{x_l}^2$	CoNet	0.040	0.043	0.047
	CPNT	0.039	0.055	0.056
$x_k = sinx_l$	CoNet	0.045	0.050	0.058
	CPNT	0.057	0.055	0.051
$x_k = sin^2x_l$	CoNet	0.046	0.061	0.055
	CPNT	0.048	0.048	0.047

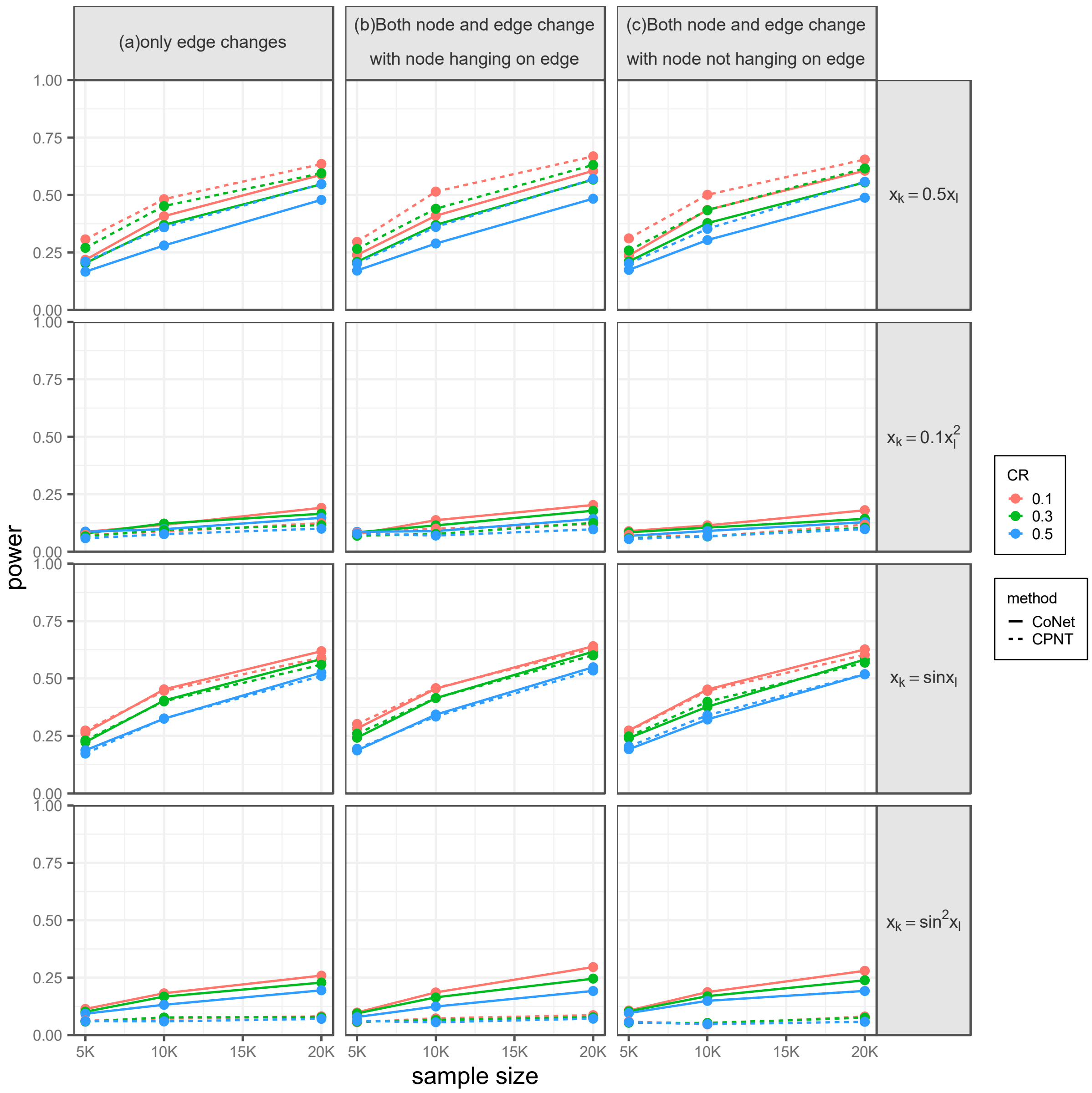

Figure S2. The power for detecting the effect of the edge on the survival phenotype under the setting that the effecting edge is randomly selected, with DPR as the gene expression prediction model in TWAS. Simulations were conducted with four different between-node correlation patterns (the recombination of quadratic and sine, sine, quadratic, and linear) and three different censoring rates (0.1, 0.3, and 0.5): (**a**) Only a node has an effect; (**b**) Both node and edge have effects, with the effecting node hanging on the edge; (**c**) Both node and edge have effects, with the effecting node not hanging on the edge.

Table S11. The type I error for detecting the effect of the node on the survival phenotype under three scenarios where the effecting node is pre-specified(n=5000), with BSLMM as the gene expression prediction model in TWAS. Simulations were conducted with four different between-node correlation patterns (the recombination of quadratic and sine, sine, quadratic, and linear) and three different censoring rates (0.1, 0.3, and 0.5).

**Scenario 1 :Only node changes**				
Correlation patterns	Methods	Censoring rate		
		0.1	0.3	0.5
$x_k = 0.5x_l$	CoNet	0.052	0.047	0.05
	CPNT	0.051	0.052	0.048
$x_k = 0.1{x_l}^2$	CoNet	0.051	0.052	0.061
	CPNT	0.050	0.053	0.063
$x_k = sinx_l$	CoNet	0.051	0.052	0.059
	CPNT	0.048	0.052	0.062
$x_k = sin^2x_l$	CoNet	0.050	0.052	0.061
	CPNT	0.049	0.053	0.064
**Scenario 2 : Both node and edge change with node hanging on edge**				
Correlation patterns	Methods	Censoring rate		
		0.1	0.3	0.5
$x_k = 0.5x_l$	CoNet	0.050	0.046	0.047
	CPNT	0.052	0.050	0.043
$x_k = 0.1{x_l}^2$	CoNet	0.052	0.054	0.051
	CPNT	0.052	0.054	0.048
$x_k = sinx_l$	CoNet	0.054	0.059	0.055
	CPNT	0.057	0.053	0.053
$x_k = sin^2x_l$	CoNet	0.052	0.048	0.047
	CPNT	0.051	0.050	0.050
**Scenario 3: Both node and edge change with node not hanging on edge**				
Correlation patterns	Methods	Censoring rate		
		0.1	0.3	0.5
$x_k = 0.5x_l$	CoNet	0.052	0.060	0.053
	CPNT	0.055	0.058	0.054
$x_k = 0.1{x_l}^2$	CoNet	0.056	0.068	0.064
	CPNT	0.056	0.064	0.061
$x_k = sinx_l$	CoNet	0.057	0.067	0.067
	CPNT	0.056	0.065	0.058
$x_k = sin^2x_l$	CoNet	0.060	0.065	0.065
	CPNT	0.054	0.065	0.058

Table S12. The type I error for detecting the effect of the node on the survival phenotype under three scenarios where the effecting node is pre-specified(n=10000), with BSLMM as the gene expression prediction model in TWAS. Simulations were conducted with four different between-node correlation patterns (the recombination of quadratic and sine, sine, quadratic, and linear) and three different censoring rates (0.1, 0.3, and 0.5).

Correlation patterns	Methods	Censoring rate		
		0.1	0.3	0.5
**Scenario 1 :Only node changes**				
$x_k = 0.5x_l$	CoNet	0.044	0.045	0.046
	CPNT	0.046	0.043	0.046
$x_k = 0.1{x_l}^2$	CoNet	0.046	0.047	0.051
	CPNT	0.046	0.044	0.050
$x_k = sinx_l$	CoNet	0.048	0.047	0.049
	CPNT	0.045	0.044	0.049
$x_k = sin^2x_l$	CoNet	0.046	0.049	0.050
	CPNT	0.045	0.045	0.051
**Scenario 2 : Both node and edge change with node hanging on edge**				
$x_k = 0.5x_l$	CoNet	0.032	0.031	0.041
	CPNT	0.036	0.036	0.041
$x_k = 0.1{x_l}^2$	CoNet	0.051	0.048	0.047
	CPNT	0.049	0.050	0.048
$x_k = sinx_l$	CoNet	0.046	0.043	0.034
	CPNT	0.042	0.041	0.034
$x_k = sin^2x_l$	CoNet	0.051	0.040	0.046
	CPNT	0.049	0.046	0.048
**Scenario 3: Both node and edge change with node not hanging on edge**				
$x_k = 0.5x_l$	CoNet	0.048	0.050	0.042
	CPNT	0.040	0.044	0.045
$x_k = 0.1{x_l}^2$	CoNet	0.050	0.060	0.055
	CPNT	0.056	0.064	0.055
$x_k = sinx_l$	CoNet	0.049	0.060	0.053
	CPNT	0.056	0.063	0.054
$x_k = sin^2x_l$	CoNet	0.047	0.060	0.055
	CPNT	0.055	0.062	0.054

Table S13. The type I error for detecting the effect of the node on the survival phenotype under three scenarios where the effecting node is pre-specified(n=20000), with BSLMM as the gene expression prediction model in TWAS. Simulations were conducted with four different between-node correlation patterns (the recombination of quadratic and sine, sine, quadratic, and linear) and three different censoring rates (0.1, 0.3, and 0.5).

**Scenario 1 :Only node changes**				
Correlation patterns	Methods	Censoring rate		
		0.1	0.3	0.5
$x_k = 0.5x_l$	CoNet	0.062	0.055	0.05
	CPNT	0.059	0.052	0.052
$x_k = 0.1{x_l}^2$	CoNet	0.044	0.056	0.039
	CPNT	0.045	0.056	0.037
$x_k = sinx_l$	CoNet	0.044	0.056	0.04
	CPNT	0.044	0.056	0.039
$x_k = sin^2x_l$	CoNet	0.043	0.057	0.040
	CPNT	0.043	0.056	0.038
**Scenario 2 : Both node and edge change with node hanging on edge**				
Correlation patterns	Methods	Censoring rate		
		0.1	0.3	0.5
$x_k = 0.5x_l$	CoNet	0.047	0.053	0.052
	CPNT	0.046	0.052	0.049
$x_k = 0.1{x_l}^2$	CoNet	0.051	0.041	0.045
	CPNT	0.045	0.048	0.045
$x_k = sinx_l$	CoNet	0.053	0.040	0.048
	CPNT	0.053	0.037	0.047
$x_k = sin^2x_l$	CoNet	0.056	0.043	0.042
	CPNT	0.048	0.052	0.044
**Scenario 3: Both node and edge change with node not hanging on edge**				
Correlation patterns	Methods	Censoring rate		
		0.1	0.3	0.5
$x_k = 0.5x_l$	CoNet	0.058	0.071	0.055
	CPNT	0.060	0.073	0.055
$x_k = 0.1{x_l}^2$	CoNet	0.051	0.053	0.052
	CPNT	0.053	0.058	0.054
$x_k = sinx_l$	CoNet	0.053	0.055	0.054
	CPNT	0.053	0.058	0.056
$x_k = sin^2x_l$	CoNet	0.050	0.053	0.053
	CPNT	0.053	0.058	0.055

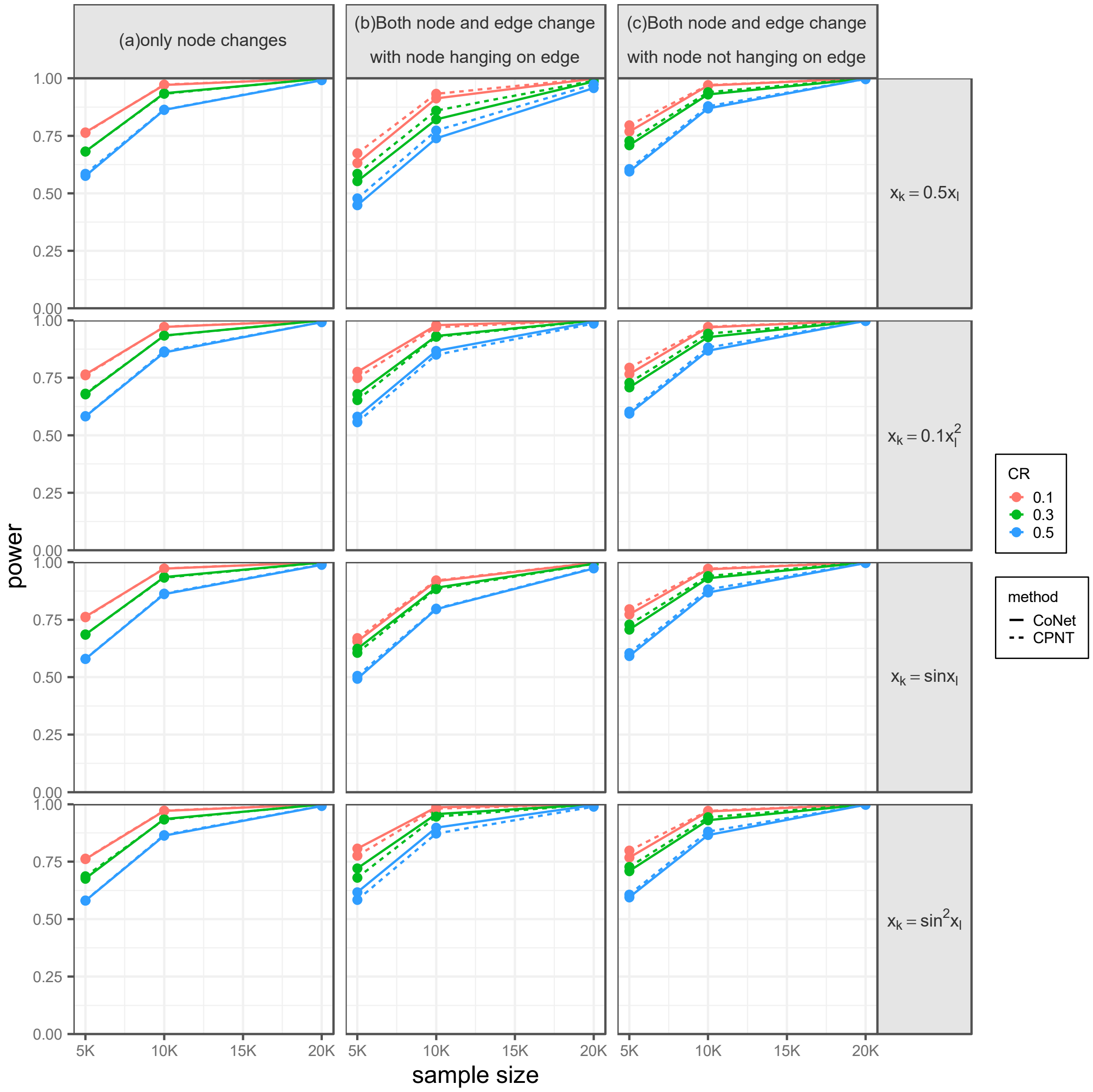

Figure S3. The power for detecting the effect of the node on the survival phenotype under the setting that the effecting node is pre-specified, with BSLMM as the gene expression prediction model in TWAS. Simulations were conducted with four different between-node correlation patterns (the recombination of quadratic and sine, sine, quadratic, and linear) and three different censoring rates (0.1, 0.3, and 0.5): (**a**) Only a node has an effect; (**b**) Both node and edge have effects, with the effecting node hanging on the edge; (**c**) Both node and edge have effects, with the effecting node not hanging on the edge.

Table S14. The type I error for detecting the effect of the edge on the survival phenotype under three scenarios where the effecting edge is pre-specified(n=5000), with BSLMM as the gene expression prediction model in TWAS. Simulations were conducted with four different between-node correlation patterns (the recombination of quadratic and sine, sine, quadratic, and linear) and three different censoring rates (0.1, 0.3, and 0.5).

**Scenario 1 :Only edge changes**				
Correlation patterns	Methods	Censoring rate		
		0.1	0.3	0.5
$x_k = 0.5x_l$	CoNet	0.046	0.061	0.057
	CPNT	0.058	0.058	0.053
$x_k = 0.1{x_l}^2$	CoNet	0.044	0.051	0.053
	CPNT	0.044	0.043	0.044
$x_k = sinx_l$	CoNet	0.039	0.044	0.043
	CPNT	0.044	0.045	0.042
$x_k = sin^2x_l$	CoNet	0.051	0.050	0.052
	CPNT	0.043	0.047	0.046
**Scenario 2 : Both node and edge change with node hanging on edge**				
Correlation patterns	Methods	Censoring rate		
		0.1	0.3	0.5
$x_k = 0.5x_l$	CoNet	0.042	0.053	0.056
	CPNT	0.043	0.051	0.059
$x_k = 0.1{x_l}^2$	CoNet	0.056	0.049	0.048
	CPNT	0.045	0.046	0.054
$x_k = sinx_l$	CoNet	0.036	0.054	0.060
	CPNT	0.044	0.061	0.053
$x_k = sin^2x_l$	CoNet	0.054	0.047	0.051
	CPNT	0.048	0.061	0.049
**Scenario 3: Both node and edge change with node not hanging on edge**				
Correlation patterns	Methods	Censoring rate		
		0.1	0.3	0.5
$x_k = 0.5x_l$	CoNet	0.053	0.049	0.045
	CPNT	0.040	0.039	0.050
$x_k = 0.1{x_l}^2$	CoNet	0.056	0.052	0.048
	CPNT	0.047	0.056	0.054
$x_k = sinx_l$	CoNet	0.059	0.059	0.060
	CPNT	0.054	0.062	0.057
$x_k = sin^2x_l$	CoNet	0.048	0.052	0.055
	CPNT	0.047	0.044	0.056

Table S15. The type I error for detecting the effect of the edge on the survival phenotype under three scenarios where the effecting edge is pre-specified(n=10000), with BSLMM as the gene expression prediction model in TWAS. Simulations were conducted with four different between-node correlation patterns (the recombination of quadratic and sine, sine, quadratic, and linear) and three different censoring rates (0.1, 0.3, and 0.5).

Scenario 1 :Only edge changes				
Correlation patterns	Methods	Censoring rate		
		0.1	0.3	0.5
$x_k = 0.5x_l$	CoNet	0.049	0.048	0.058
	CPNT	0.052	0.053	0.056
$x_k = 0.1{x_l}^2$	CoNet	0.056	0.057	0.050
	CPNT	0.053	0.049	0.044
$x_k = sinx_l$	CoNet	0.048	0.051	0.044
	CPNT	0.042	0.056	0.041
$x_k = sin^2x_l$	CoNet	0.045	0.043	0.051
	CPNT	0.050	0.042	0.043
**Scenario 2 : Both node and edge change with node hanging on edge**				
Correlation patterns	Methods	Censoring rate		
		0.1	0.3	0.5
$x_k = 0.5x_l$	CoNet	0.046	0.055	0.047
	CPNT	0.057	0.063	0.055
$x_k = 0.1{x_l}^2$	CoNet	0.058	0.041	0.060
	CPNT	0.055	0.036	0.054
$x_k = sinx_l$	CoNet	0.056	0.042	0.058
	CPNT	0.051	0.044	0.066
$x_k = sin^2x_l$	CoNet	0.050	0.047	0.046
	CPNT	0.053	0.058	0.051
**Scenario 3: Both node and edge change with node not hanging on edge**				
Correlation patterns	Methods	Censoring rate		
		0.1	0.3	0.5
$x_k = 0.5x_l$	CoNet	0.061	0.060	0.061
	CPNT	0.056	0.054	0.051
$x_k = 0.1{x_l}^2$	CoNet	0.056	0.053	0.044
	CPNT	0.048	0.040	0.048
$x_k = sinx_l$	CoNet	0.047	0.056	0.052
	CPNT	0.047	0.040	0.055
$x_k = sin^2x_l$	CoNet	0.058	0.059	0.049
	CPNT	0.036	0.040	0.042

Table S16. The type I error for detecting the effect of the edge on the survival phenotype under three scenarios where the effecting edge is pre-specified(n=20000), with BSLMM as the gene expression prediction model in TWAS. Simulations were conducted with four different between-node correlation patterns (the recombination of quadratic and sine, sine, quadratic, and linear) and three different censoring rates (0.1, 0.3, and 0.5).

**Scenario 1 :Only edge changes**				
Correlation patterns	Methods	Censoring rate		
		0.1	0.3	0.5
$x_k = 0.5x_l$	CoNet	0.049	0.040	0.054
	CPNT	0.049	0.052	0.064
$x_k = 0.1{x_l}^2$	CoNet	0..058	0.051	0.060
	CPNT	0.057	0.047	0.050
$x_k = sinx_l$	CoNet	0.049	0.046	0.039
	CPNT	0.047	0.047	0.045
$x_k = sin^2x_l$	CoNet	0.044	0.049	0.052
	CPNT	0.044	0.046	0.044
**Scenario 2 : Both node and edge change with node hanging on edge**				
Correlation patterns	Methods	Censoring rate		
		0.1	0.3	0.5
$x_k = 0.5x_l$	CoNet	0.060	0.044	0.052
	CPNT	0.061	0.050	0.044
$x_k = 0.1{x_l}^2$	CoNet	0.048	0.066	0.058
	CPNT	0.042	0.068	0.051
$x_k = sinx_l$	CoNet	0.045	0.045	0.041
	CPNT	0.051	0.045	0.046
$x_k = sin^2x_l$	CoNet	0.059	0.057	0.038
	CPNT	0.061	0.046	0.045
**Scenario 3: Both node and edge change with node not hanging on edge**				
Correlation patterns	Methods	Censoring rate		
		0.1	0.3	0.5
$x_k = 0.5x_l$	CoNet	0.051	0.059	0.052
	CPNT	0.044	0.042	0.048
$x_k = 0.1{x_l}^2$	CoNet	0.043	0.041	0.047
	CPNT	0.053	0.051	0.061
$x_k = sinx_l$	CoNet	0.055	0.053	0.050
	CPNT	0.050	0.058	0.051
$x_k = sin^2x_l$	CoNet	0.053	0.055	0.055
	CPNT	0.062	0.057	0.053

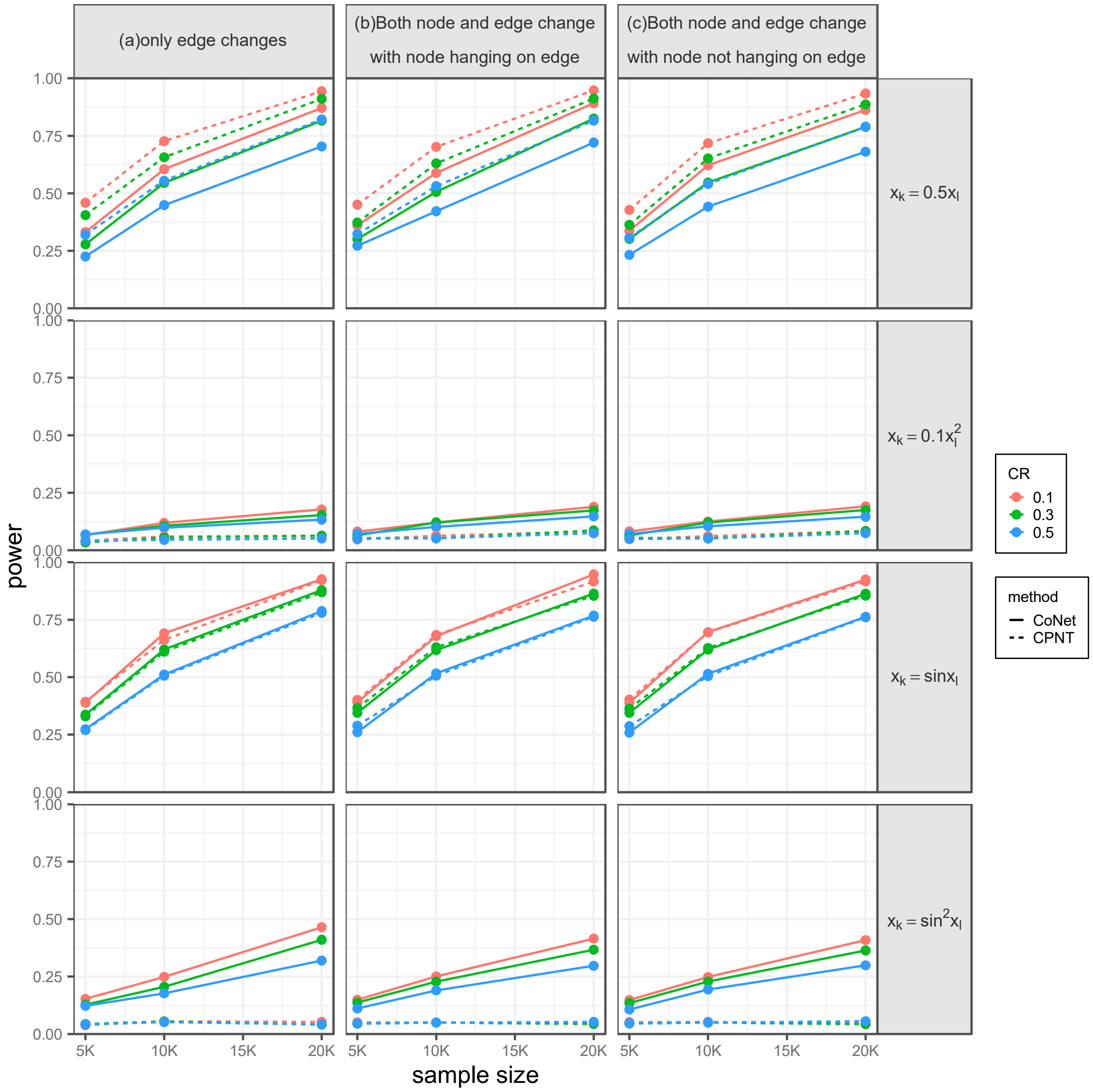

Figure S4. The power for detecting the effect of the edge on the survival phenotype under the setting that the effecting edge is pre-specified, with BSLMM as the gene expression prediction model in TWAS. Simulations were conducted with four different between-node correlation patterns (the recombination of quadratic and sine, sine, quadratic, and linear) and three different censoring rates (0.1, 0.3, and 0.5): (**a**) Only a node has an effect; (**b**) Both node and edge have effects, with the effecting node hanging on the edge; (**c**) Both node and edge have effects, with the effecting node not hanging on the edge.

Table S17. The type I error for detecting the effect of the node on the survival phenotype under three scenarios where the effecting node is randomly selected (n=5000), with BSLMM as the gene expression prediction model in TWAS. Simulations were conducted with four different between-node correlation patterns (the recombination of quadratic and sine, sine, quadratic, and linear) and three different censoring rates (0.1, 0.3, and 0.5).

**Scenario 1 :Only node changes**				
Correlation patterns	Methods	Censoring rate		
		0.1	0.3	0.5
$x_k = 0.5x_l$	CoNet	0.048	0.044	0.046
	CPNT	0.047	0.039	0.041
$x_k = 0.1{x_l}^2$	CoNet	0.049	0.041	0.047
	CPNT	0.046	0.044	0.050
$x_k = sinx_l$	CoNet	0.048	0.040	0.047
	CPNT	0.041	0.043	0.049
$x_k = sin^2x_l$	CoNet	0.047	0.037	0.048
	CPNT	0.042	0.043	0.050
**Scenario 2 : Both node and edge change with node hanging on edge**				
Correlation patterns	Methods	Censoring rate		
		0.1	0.3	0.5
$x_k = 0.5x_l$	CoNet	0.046	0.058	0.061
	CPNT	0.045	0.060	0.056
$x_k = 0.1{x_l}^2$	CoNet	0.045	0.041	0.064
	CPNT	0.044	0.044	0.064
$x_k = sinx_l$	CoNet	0.041	0.043	0.067
	CPNT	0.031	0.049	0.064
$x_k = sin^2x_l$	CoNet	0.037	0.049	0.062
	CPNT	0.032	0.049	0.062
**Scenario 3: Both node and edge change with node not hanging on edge**				
Correlation patterns	Methods	Censoring rate		
		0.1	0.3	0.5
$x_k = 0.5x_l$	CoNet	0.054	0.049	0.049
	CPNT	0.051	0.049	0.042
$x_k = 0.1{x_l}^2$	CoNet	0.049	0.047	0.058
	CPNT	0.045	0.045	0.060
$x_k = sinx_l$	CoNet	0.046	0.054	0.060
	CPNT	0.049	0.050	0.060
$x_k = sin^2x_l$	CoNet	0.045	0.052	0.062
	CPNT	0.048	0.048	0.058

Table S18. The type I error for detecting the effect of the node on the survival phenotype under three scenarios where the effecting node is randomly selected (n=10000), with BSLMM as the gene expression prediction model in TWAS. Simulations were conducted with four different between-node correlation patterns (the recombination of quadratic and sine, sine, quadratic, and linear) and three different censoring rates (0.1, 0.3, and 0.5).

Correlation patterns	Methods	Censoring rate 0.1	Censoring rate 0.3	Censoring rate 0.5
**Scenario 1 :Only node changes**				
$x_k = 0.5x_l$	CoNet	0.035	0.044	0.047
	CPNT	0.037	0.042	0.047
$x_k = 0.1{x_l}^2$	CoNet	0.040	0.045	0.045
	CPNT	0.039	0.046	0.045
$x_k = sinx_l$	CoNet	0.047	0.042	0.045
	CPNT	0.037	0.048	0.046
$x_k = sin^2x_l$	CoNet	0.045	0.044	0.044
	CPNT	0.038	0.049	0.046
**Scenario 2 : Both node and edge change with node hanging on edge**				
$x_k = 0.5x_l$	CoNet	0.039	0.051	0.051
	CPNT	0.037	0.050	0.048
$x_k = 0.1{x_l}^2$	CoNet	0.046	0.042	0.047
	CPNT	0.042	0.039	0.042
$x_k = sinx_l$	CoNet	0.042	0.039	0.049
	CPNT	0.038	0.039	0.043
$x_k = sin^2x_l$	CoNet	0.043	0.037	0.040
	CPNT	0.038	0.039	0.043
**Scenario 3: Both node and edge change with node not hanging on edge**				
$x_k = 0.5x_l$	CoNet	0.044	0.046	0.044
	CPNT	0.043	0.047	0.042
$x_k = 0.1{x_l}^2$	CoNet	0.039	0.042	0.038
	CPNT	0.037	0.042	0.039
$x_k = sinx_l$	CoNet	0.034	0.041	0.037
	CPNT	0.033	0.042	0.040
$x_k = sin^2x_l$	CoNet	0.034	0.043	0.039
	CPNT	0.033	0.042	0.039

Table S19. The type I error for detecting the effect of the node on the survival phenotype under three scenarios where the effecting node is randomly selected (n=20000), with BSLMM as the gene expression prediction model in TWAS. Simulations were conducted with four different between-node correlation patterns (the recombination of quadratic and sine, sine, quadratic, and linear) and three different censoring rates (0.1, 0.3, and 0.5).

Correlation patterns	Methods	Censoring rate		
**Scenario 1 :Only node changes**				
Correlation patterns	Methods	0.1	0.3	0.5
$x_k = 0.5x_l$	CoNet	0.043	0.040	0.039
	CPNT	0.045	0.040	0.043
$x_k = 0.1{x_l}^2$	CoNet	0.055	0.055	0.049
	CPNT	0.057	0.055	0.050
$x_k = sinx_l$	CoNet	0.040	0.053	0.048
	CPNT	0.042	0.051	0.050
$x_k = sin^2x_l$	CoNet	0.040	0.052	0.051
	CPNT	0.044	0.051	0.051
**Scenario 2 : Both node and edge change with node hanging on edge**				
Correlation patterns	Methods	0.1	0.3	0.5
$x_k = 0.5x_l$	CoNet	0.051	0.039	0.052
	CPNT	0.046	0.042	0.049
$x_k = 0.1{x_l}^2$	CoNet	0.025	0.034	0.036
	CPNT	0.032	0.036	0.036
$x_k = sinx_l$	CoNet	0.029	0.036	0.037
	CPNT	0.028	0.036	0.035
$x_k = sin^2x_l$	CoNet	0.029	0.037	0.037
	CPNT	0.028	0.035	0.035
**Scenario 3: Both node and edge change with node not hanging on edge**				
Correlation patterns	Methods	0.1	0.3	0.5
$x_k = 0.5x_l$	CoNet	0.036	0.040	0.041
	CPNT	0.041	0.044	0.044
$x_k = 0.1{x_l}^2$	CoNet	0.062	0.050	0.060
	CPNT	0.063	0.055	0.060
$x_k = sinx_l$	CoNet	0.060	0.056	0.061
	CPNT	0.059	0.057	0.060
$x_k = sin^2x_l$	CoNet	0.058	0.056	0.060
	CPNT	0.059	0.054	0.060

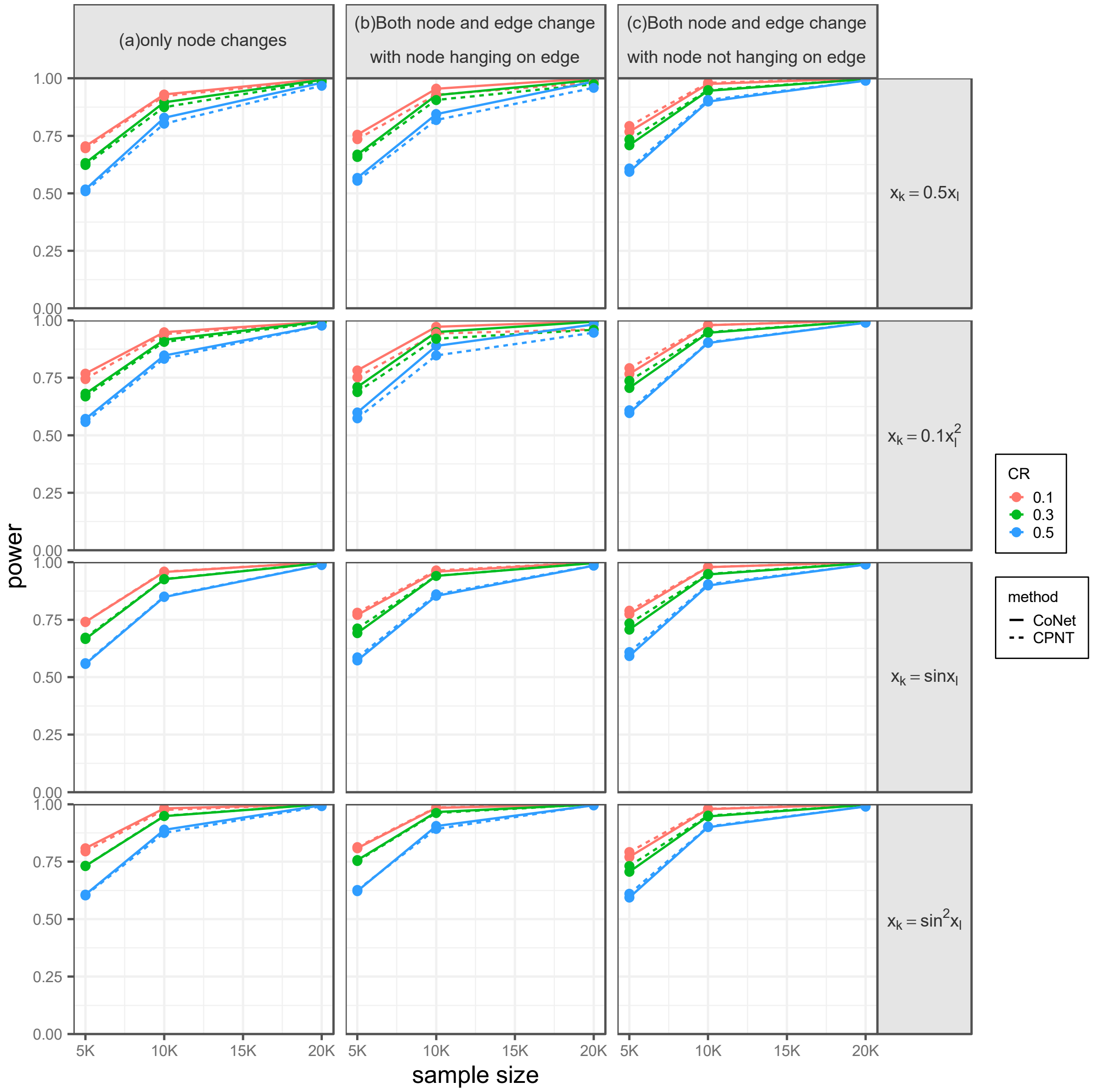

Figure S5. The power for detecting the effect of the node on the survival phenotype under the setting that the effecting node is randomly selected, with BSLMM as the gene expression prediction model in TWAS. Simulations were conducted with four different between-node correlation patterns (the recombination of quadratic and sine, sine, quadratic, and linear) and three different censoring rates (0.1, 0.3, and 0.5): (**a**) Only a node has an effect; (**b**) Both node and edge have effects, with the effecting node hanging on the edge; (**c**) Both node and edge have effects, with the effecting node not hanging on the edge.

Table S20. The type I error for detecting the effect of the edge on the survival phenotype under three scenarios where the effecting edge is randomly selected (n=5000), with BSLMM as the gene expression prediction model in TWAS. Simulations were conducted with four different between-node correlation patterns (the recombination of quadratic and sine, sine, quadratic, and linear) and three different censoring rates (0.1, 0.3, and 0.5).

Correlation patterns	Methods	Censoring rate		
		0.1	0.3	0.5
**Scenario 1 :Only edge changes**				
$x_k = 0.5x_l$	CoNet	0.048	0.062	0.058
	CPNT	0.048	0.059	0.052
$x_k = 0.1{x_l}^2$	CoNet	0.050	0.057	0.048
	CPNT	0.058	0.051	0.051
$x_k = sinx_l$	CoNet	0.043	0.051	0.046
	CPNT	0.052	0.052	0.055
$x_k = sin^2x_l$	CoNet	0.051	0.051	0.050
	CPNT	0.052	0.048	0.050
**Scenario 2 : Both node and edge change with node hanging on edge**				
$x_k = 0.5x_l$	CoNet	0.041	0.056	0.061
	CPNT	0.051	0.041	0.052
$x_k = 0.1{x_l}^2$	CoNet	0.046	0.046	0.049
	CPNT	0.052	0.053	0.049
$x_k = sinx_l$	CoNet	0.042	0.050	0.051
	CPNT	0.046	0.050	0.046
$x_k = sin^2x_l$	CoNet	0.044	0.050	0.048
	CPNT	0.049	0.052	0.045
**Scenario 3: Both node and edge change with node not hanging on edge**				
$x_k = 0.5x_l$	CoNet	0.043	0.045	0.046
	CPNT	0.043	0.050	0.051
$x_k = 0.1{x_l}^2$	CoNet	0.056	0.053	0.054
	CPNT	0.048	0.048	0.042
$x_k = sinx_l$	CoNet	0.038	0.046	0.053
	CPNT	0.037	0.047	0.046
$x_k = sin^2x_l$	CoNet	0.038	0.042	0.052
	CPNT	0.033	0.046	0.043

Table S21. The type I error for detecting the effect of the edge on the survival phenotype under three scenarios where the effecting edge is randomly selected (n=10000), with BSLMM as the gene expression prediction model in TWAS. Simulations were conducted with four different between-node correlation patterns (the recombination of quadratic and sine, sine, quadratic, and linear) and three different censoring rates (0.1, 0.3, and 0.5).

Correlation patterns	Methods	Censoring rate		
**Scenario 1 :Only edge changes**				
		0.1	0.3	0.5
$x_k = 0.5x_l$	CoNet	0.027	0.042	0.038
	CPNT	0.048	0.056	0.057
$x_k = 0.1{x_l}^2$	CoNet	0.050	0.048	0.042
	CPNT	0.046	0.044	0.054
$x_k = sinx_l$	CoNet	0.047	0.051	0.044
	CPNT	0.051	0.051	0.054
$x_k = sin^2x_l$	CoNet	0.042	0.043	0.040
	CPNT	0.044	0.045	0.051
**Scenario 2 : Both node and edge change with node hanging on edge**				
Correlation patterns	Methods	Censoring rate		
		0.1	0.3	0.5
$x_k = 0.5x_l$	CoNet	0.049	0.055	0.050
	CPNT	0.054	0.058	0.047
$x_k = 0.1{x_l}^2$	CoNet	0.045	0.037	0.051
	CPNT	0.061	0.059	0.060
$x_k = sinx_l$	CoNet	0.047	0.044	0.042
	CPNT	0.056	0.055	0.056
$x_k = sin^2x_l$	CoNet	0.045	0.040	0.045
	CPNT	0.057	0.056	0.058
**Scenario 3: Both node and edge change with node not hanging on edge**				
Correlation patterns	Methods	Censoring rate		
		0.1	0.3	0.5
$x_k = 0.5x_l$	CoNet	0.040	0.061	0.056
	CPNT	0.040	0.042	0.040
$x_k = 0.1{x_l}^2$	CoNet	0.049	0.045	0.048
	CPNT	0.046	0.048	0.047
$x_k = sinx_l$	CoNet	0.040	0.052	0.043
	CPNT	0.037	0.046	0.050
$x_k = sin^2x_l$	CoNet	0.036	0.045	0.043
	CPNT	0.043	0.053	0.052

Table S22. The type I error for detecting the effect of the edge on the survival phenotype under three scenarios where the effecting edge is randomly selected (n=20000), with BSLMM as the gene expression prediction model in TWAS. Simulations were conducted with four different between-node correlation patterns (the recombination of quadratic and sine, sine, quadratic, and linear) and three different censoring rates (0.1, 0.3, and 0.5).

**Scenario 1 :Only edge changes**				
Correlation patterns	Methods	Censoring rate		
		0.1	0.3	0.5
$x_k = 0.5x_l$	CoNet	0.052	0.051	0.059
	CPNT	0.036	0.036	0.040
$x_k = 0.1{x_l}^2$	CoNet	0.049	0.049	0.044
	CPNT	0.041	0.047	0.056
$x_k = sinx_l$	CoNet	0.051	0.051	0.049
	CPNT	0.043	0.036	0.051
$x_k = sin^2x_l$	CoNet	0.042	0.047	0.048
	CPNT	0.048	0.048	0.055
**Scenario 2 : Both node and edge change with node hanging on edge**				
Correlation patterns	Methods	Censoring rate		
		0.1	0.3	0.5
$x_k = 0.5x_l$	CoNet	0.045	0.047	0.052
	CPNT	0.045	0.071	0.067
$x_k = 0.1{x_l}^2$	CoNet	0.045	0.049	0.048
	CPNT	0.051	0.040	0.047
$x_k = sinx_l$	CoNet	0.041	0.056	0.058
	CPNT	0.046	0.041	0.046
$x_k = sin^2x_l$	CoNet	0.040	0.049	0.047
	CPNT	0.043	0.040	0.045
**Scenario 3: Both node and edge change with node not hanging on edge**				
Correlation patterns	Methods	Censoring rate		
		0.1	0.3	0.5
$x_k = 0.5x_l$	CoNet	0.040	0.054	0.052
	CPNT	0.045	0.050	0.055
$x_k = 0.1{x_l}^2$	CoNet	0.046	0.052	0.057
	CPNT	0.054	0.052	0.066
$x_k = sinx_l$	CoNet	0.051	0.054	0.061
	CPNT	0.052	0.057	0.065
$x_k = sin^2x_l$	CoNet	0.048	0.051	0.063
	CPNT	0.044	0.049	0.063

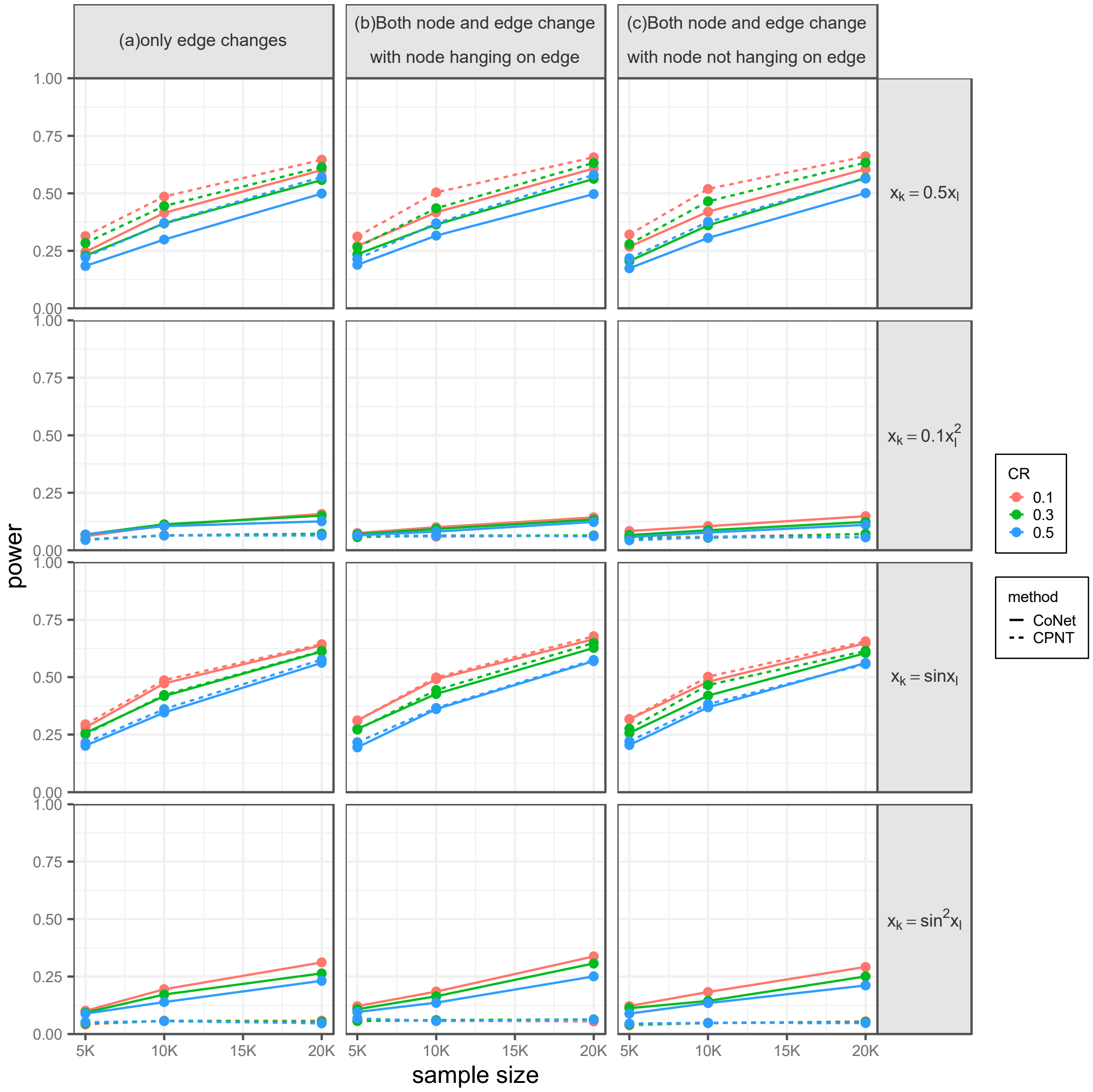

Figure S6. The power for detecting the effect of the edge on the survival phenotype under the setting that the effecting edge is randomly selected, with BSLMM as the gene expression prediction model in TWAS. Simulations were conducted with four different between-node correlation patterns (the recombination of quadratic and sine, sine, quadratic, and linear) and three different censoring rates (0.1, 0.3, and 0.5): (**a**) Only a node has an effect; (**b**) Both node and edge have effects, with the effecting node hanging on the edge; (**c**) Both node and edge have effects, with the effecting node not hanging on the edge.

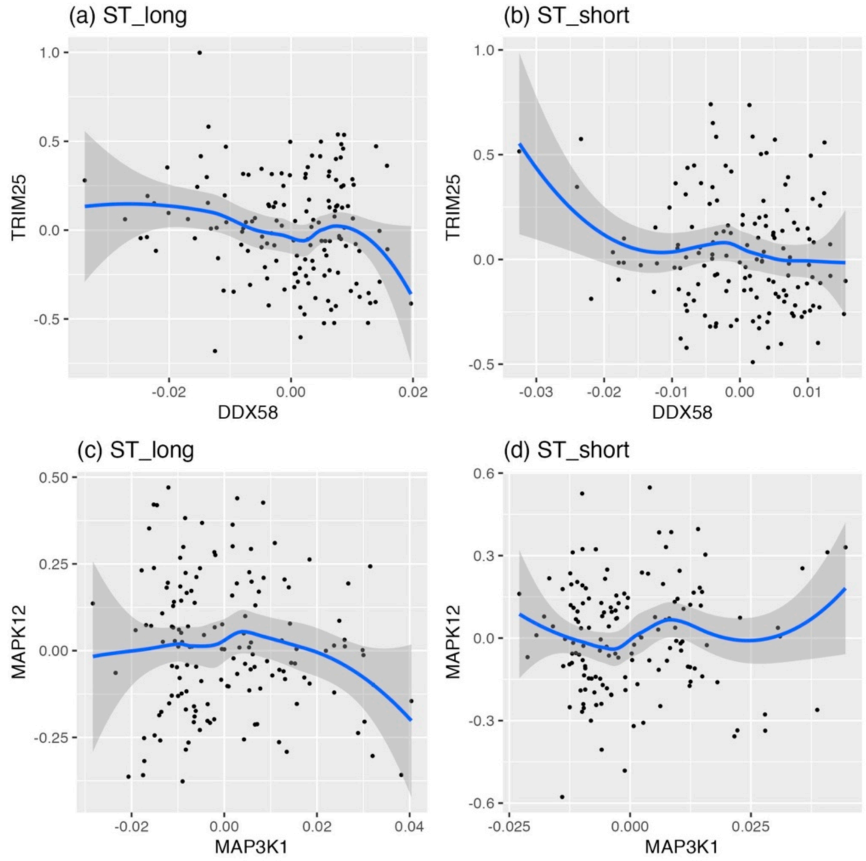

Figure S7. Scatter plot of the expression of two different genes with different survival status. The relationship between the expression of DDX58 and TRIM25 under long survival time (a) and short survival time (b). The relationship between the expression of MAP3K1 and MAPK12 under the long survival time (c) and short survival time(d).

Table S23. The type I error for testing the effect of the node and edge on the survival phenotype where the effecting nodes and the effecting edges are pre-specified(n=5000, 10000, 20000), with SNPs effect obtained from DPR model. Simulations were conducted with two different between-node correlation patterns (linear and the combination of sine and quadratic) and the censoring rate being 0.7.

**Scenario 1: Only node changes**				
Correlation patterns	Methods	Sample size		
		5000	10000	20000
$x_k = 0.5x_l$	CoNet	0.049	0.053	0.051
	CPNT	0.050	0.054	0.056
$x_k = sin^2 x_l$	CoNet	0.047	0.056	0.051
	CPNT	0.050	0.054	0.054
**Scenario 2: Only edge changes**				
Correlation patterns	Methods	Sample size		
		5000	10000	20000
$x_k = 0.5x_l$	CoNet	0.048	0.052	0.058
	CPNT	0.049	0.047	0.048

$x_k = sin^2 x_l$	CoNet	0.043	0.055	0.045
	CPNT	0.056	0.058	0.041

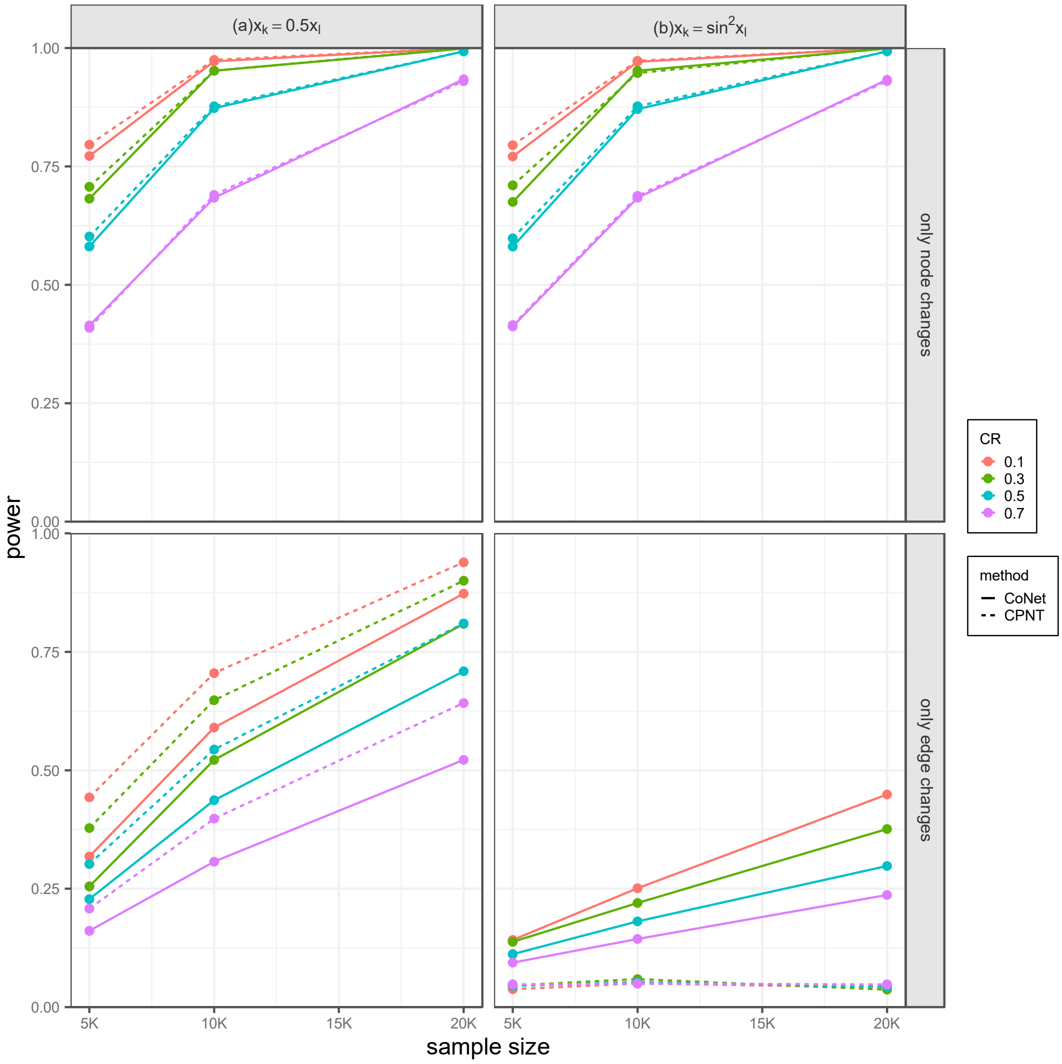

Figure S8. The power for testing the effect of the node and edge on the survival phenotype where the effecting nodes and effecting edges are pre-specified(n=5000, 10000, 20000), with SNPs effect obtained from DPR model. Simulations were conducted with two different between-node correlation patterns (linear and the combination of sine and quadratic) and four censoring rates (0.1, 0.3, 0.5 and 0.7).

Table S24. The type I error for testing the effect of the node and edge on the survival phenotype under three scenarios where the effecting nodes and the effecting edges are randomly selected(n=5000, 10000, 20000), with SNPs effect obtained from DPR model. Simulations were conducted with two different between-node correlation patterns (linear and the combination of sine and quadratic) and the censoring rate being 0.7.

**Scenario 1: Only node changes**				
Correlation patterns	Methods	Sample size		
		5000	10000	20000
$x_k = 0.5x_l$	CoNet	0.060	0.039	0.043
	CPNT	0.053	0.040	0.042
$x_k = sin^2 x_l$	CoNet	0.059	0.039	0.043
	CPNT	0.053	0.040	0.044
**Scenario 2: Only edge changes**				
Correlation patterns	Methods	Sample size		
		5000	10000	20000
$x_k = 0.5x_l$	CoNet	0.054	0.048	0.052
	CPNT	0.041	0.054	0.044
$x_k = sin^2 x_l$	CoNet	0.051	0.046	0.049
	CPNT	0.037	0.055	0.049

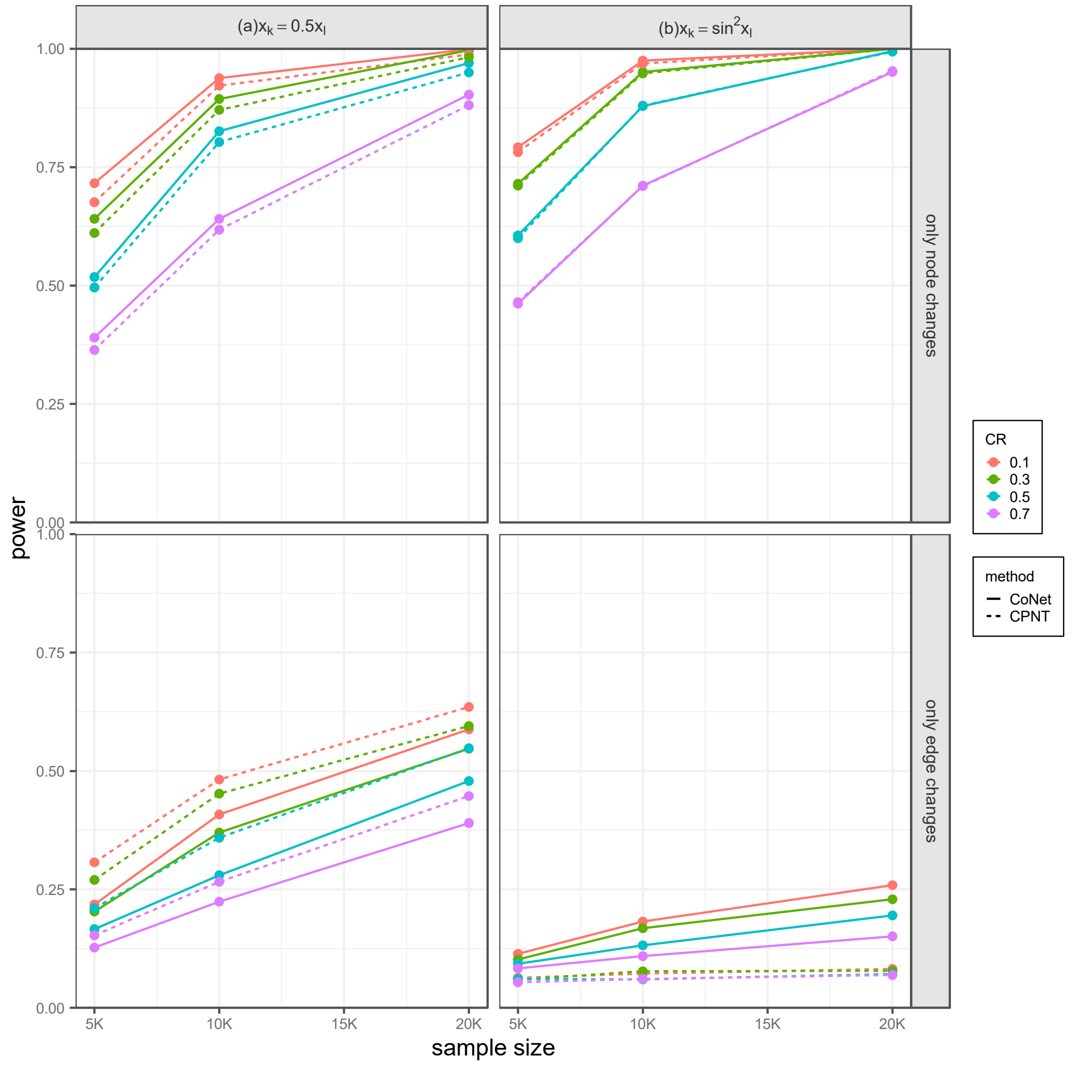

Figure S9. The power for testing the effect of the node and edge on the survival phenotype under two scenarios where the effecting nodes and the effecting edges are randomly selected (n=5000, 10000, 20000), with SNPs effect obtained from DPR model. Simulations were conducted with two different between-node correlation patterns (linear and the combination of sine and quadratic) and four censoring rates (0.1, 0.3, 0.5 and 0.7).

Table S25. Mean computational time (seconds) of both methods.

Sample size	CoNet_fix	CPNT_fix	CoNet_random	CPNT_random
5000	6.70	5.45	6.63	5.21
10000	8.14	7.91	7.62	7.38
20000	8.28	8.08	8.03	7.89
300000	20.88	12.48	20.81	12.69

Computation is carried out on a single thread of an Intel Xeon E5-2697 v3 CPU. The computational time is averaged across 20 replicates and calculated when the effecting nodes and the effecting edges are pre-specified (CoNet_fix and CPNT_fix) or

randomly selected(CoNet_random and CPNT_random). Both methods have almost the same physical memories (less than 1GB) under various situations.

Table S26. Type I error of CoNet in a reduced gene network with some proportions(0, 20%, 30%) of genes being unavailable, where the effecting nodes and effecting edges are pre-specified. Simulations were conducted under sample size 5000 with two different between-node correlation patterns, including linear relationship and nonlinear relationship (the recombination of quadratic and sine relationship) and four different censoring rates (0.1, 0.3, 0.5, 0.7).

**Scenario 1: Only node changes**					
Correlation patterns	proportion	Censoring rates			
		0.1	0.3	0.5	0.7
$x_k = 0.5x_l$	0	0.051	0.050	0.046	0.049
	20%	0.050	0.064	0.061	0.057
	30%	0.035	0.044	0.049	0.044
$x_k = sin^2 x_l$	0	0.037	0.045	0.043	0.042
	20%	0.051	0.051	0.046	0.038
	30%	0.034	0.045	0.041	0.045
**Scenario 2: Only edge changes**					
Correlation patterns	proportion	Censoring rates			
		0.1	0.3	0.5	0.7
$x_k = 0.5x_l$	0	0.049	0.039	0.043	0.048
	20%	0.053	0.053	0.051	0.057
	30%	0.041	0.056	0.049	0.044
$x_k = sin^2 x_l$	0	0.050	0.050	0.042	0.043
	20%	0.051	0.051	0.046	0.038
	30%	0.048	0.054	0.045	0.041

Table S27. Type I error of CoNet in a reduced gene network with some proportions(0, 20%, 30%) of genes being unavailable, where the effecting nodes and effecting edges are pre-specified. Simulations were conducted under sample size 10000 with two different between-node correlation patterns, including linear relationship and nonlinear relationship (the recombination of quadratic and sine relationship) and four different censoring rates (0.1, 0.3, 0.5, 0.7).

**Scenario 1: Only node changes**					
Correlation patterns	proportion	Censoring rates			
		0.1	0.3	0.5	0.7
$x_k = 0.5x_l$	0	0.047	0.052	0.057	0.053
	20%	0.054	0.056	0.049	0.060
	30%	0.058	0.049	0.053	0.050
$x_k = sin^2 x_l$	0	0.042	0.050	0.061	0.043
	20%	0.054	0.051	0.048	0.046
	30%	0.044	0.049	0.059	0.053
**Scenario 2: Only edge changes**					
Correlation patterns	proportion	Censoring rates			
		0.1	0.3	0.5	0.7
$x_k = 0.5x_l$	0	0.040	0.040	0.030	0.052

	20%	0.046	0.045	0.055	0.055
	30%	0.060	0.060	0.053	0.057
$x_k = sin^2 x_l$	0	0.062	0.058	0.069	0.055
	20%	0.054	0.051	0.048	0.046
	30%	0.066	0.056	0.067	0.051

Table S28. Type I error of CoNet in a reduced gene network with some proportions(0, 20%, 30%) of genes being unavailable, where the effecting nodes and effecting edges are pre-specified. Simulations were conducted under sample size 20000 with two different between-node correlation patterns, including linear relationship and nonlinear relationship (the recombination of quadratic and sine relationship) and four different censoring rates (0.1, 0.3, 0.5, 0.7).

**Scenario 1: Only node changes**					
Correlation patterns	proportion	Censoring rates			
		0.1	0.3	0.5	0.7
$x_k = 0.5x_l$	0	0.051	0.055	0.048	0.051
	20%	0.055	0.046	0.047	0.046
	30%	0.056	0.055	0.049	0.050
$x_k = sin^2 x_l$	0	0.055	0.057	0.053	0.045
	20%	0.051	0.048	0.050	0.045
	30%	0.056	0.056	0.051	0.051
**Scenario 2: Only edge changes**					
Correlation patterns	proportion	Censoring rates			
		0.1	0.3	0.5	0.7
$x_k = 0.5x_l$	0	0.052	0.053	0.060	0.058
	20%	0.055	0.057	0.049	0.051
	30%	0.058	0.056	0.049	0.059
$x_k = sin^2 x_l$	0	0.046	0.049	0.045	0.045
	20%	0.051	0.048	0.050	0.045
	30%	0.040	0.045	0.050	0.051

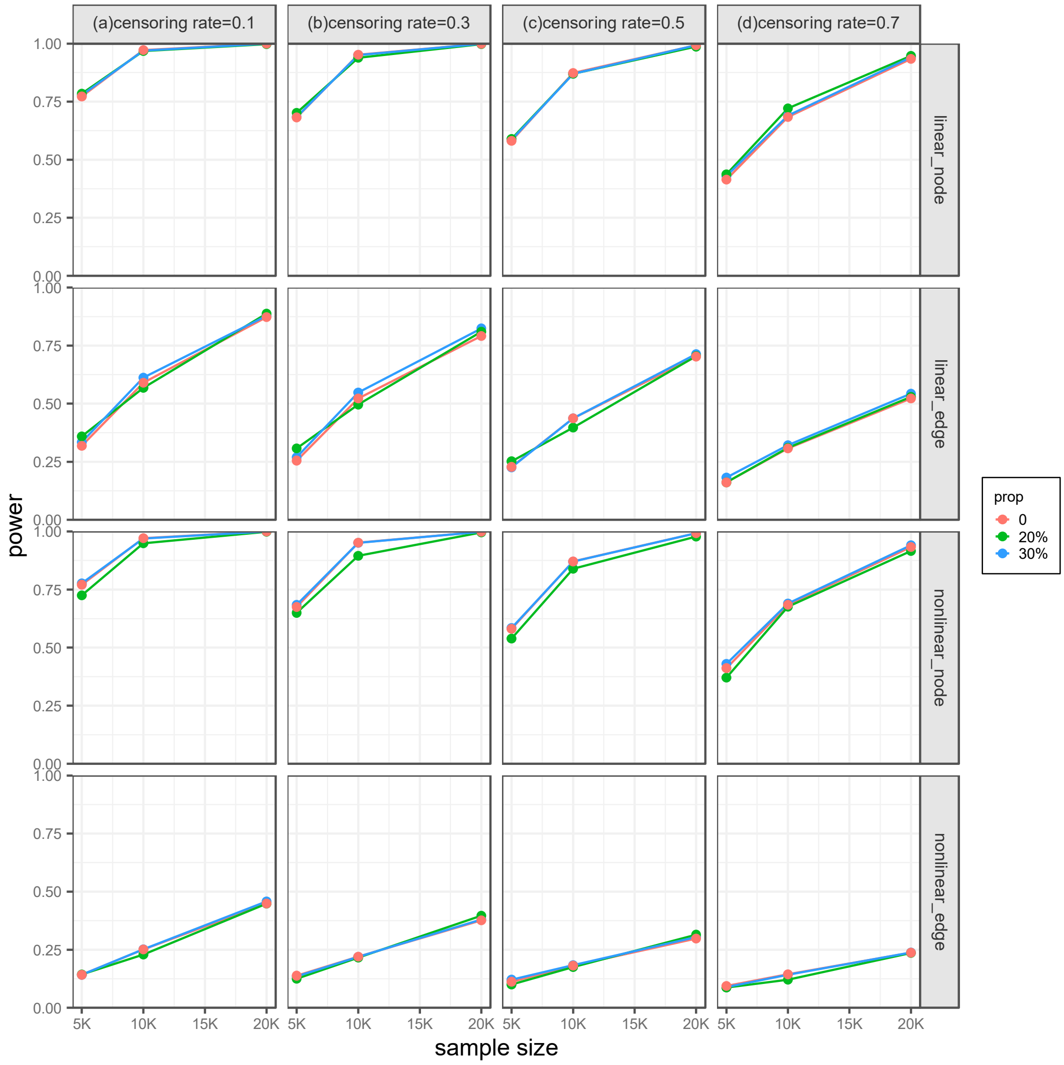

Figure S10. Power of CoNet in a reduced gene network with some genes being unavailable, where the effecting nodes and effecting edges are pre-specified. Power (y-axis) at a significance level 0.05 is plotted against different sample size (x-axis). The proportions of "20%" and "30%" represent 20% and 30% genes are unavailable in network, "0" means the genes are all available. Various settings are displayed from top to bottom, including the power for detecting the effect of node and the effect of edge in linear relationship as well as in nonlinear (the recombination of quadratic and sine relationship) relationship. Different censoring rates are listed from left to right, including (**a**) 0.1, (**b**) 0.3, (**c**) 0.5 and (**d**) 0.7.

Table S29. Type I error of CoNet in a reduced gene network with some proportions(0, 20%, 30%) of genes being unavailable, where the effecting nodes and effecting edges are randomly selected. Simulations were conducted under sample size 5000 with two different between-node correlation patterns, including linear relationship and nonlinear relationship (the recombination of quadratic and sine relationship) and four different censoring rates (0.1, 0.3, 0.5, 0.7).

Scenario 1: Only node changes					
Correlation patterns	proportion	Censoring rates			
		0.1	0.3	0.5	0.7
$x_k = 0.5x_l$	0	0.049	0.054	0.047	0.041
	20%	0.054	0.051	0.054	0.053
	30%	0.062	0.060	0.057	0.051
$x_k = sin^2 x_l$	0	0.046	0.052	0.048	0.037
	20%	0.058	0.054	0.055	0.053
	30%	0.060	0.058	0.062	0.053
**Scenario 2: Only edge changes**					
Correlation patterns	proportion	Censoring rates			
		0.1	0.3	0.5	0.7
$x_k = 0.5x_l$	0	0.041	0.043	0.053	0.054
	20%	0.072	0.058	0.059	0.054
	30%	0.048	0.050	0.059	0.060
$x_k = sin^2 x_l$	0	0.039	0.051	0.048	0.051
	20%	0.071	0.063	0.054	0.054
	30%	0.047	0.049	0.058	0.056

Table S30. Type I error of CoNet in a reduced gene network with some proportions(0, 20%, 30%) of genes being unavailable, where the effecting nodes and effecting edges are randomly selected. Simulations were conducted under sample size 10000 with two different between-node correlation patterns, including linear relationship and nonlinear relationship (the recombination of quadratic and sine relationship) and four different censoring rates (0.1, 0.3, 0.5, 0.7).

Scenario 1: Only node changes					
Correlation patterns	proportion	Censoring rates			
		0.1	0.3	0.5	0.7
$x_k = 0.5x_l$	0	0.036	0.047	0.054	0.048
	20%	0.053	0.053	0.057	0.058
	30%	0.049	0.050	0.054	0.059
$x_k = sin^2 x_l$	0	0.062	0.044	0.035	0.055
	20%	0.054	0.050	0.054	0.057
	30%	0.051	0.051	0.054	0.059
**Scenario 2: Only edge changes**					
Correlation patterns	proportion	Censoring rates			
		0.1	0.3	0.5	0.7
$x_k = 0.5x_l$	0	0.047	0.058	0.068	0.048
	20%	0.040	0.042	0.053	0.049
	30%	0.046	0.036	0.050	0.038
$x_k = sin^2 x_l$	0	0.028	0.045	0.047	0.046

	20%	0.045	0.045	0.045	0.049
	30%	0.058	0.044	0.048	0.040

Table S31. Type I error of CoNet in a reduced gene network with some proportions(0, 20%, 30%) of genes being unavailable, where the effecting nodes and effecting edges are randomly selected. Simulations were conducted under sample size 20000 with two different between-node correlation patterns, including linear relationship and nonlinear relationship (the recombination of quadratic and sine relationship) and four different censoring rates (0.1, 0.3, 0.5, 0.7).

**Scenario 1: Only node changes**					
Correlation patterns	proportion	Censoring rates			
		0.1	0.3	0.5	0.7
$x_k = 0.5x_l$	0	0.056	0.056	0.053	0.052
	20%	0.045	0.059	0.055	0.045
	30%	0.059	0.051	0.059	0.047
$x_k = sin^2 x_l$	0	0.047	0.061	0.048	0.049
	20%	0.047	0.060	0.056	0.046
	30%	0.057	0.061	0.058	0.044
**Scenario 2: Only edge changes**					
Correlation patterns	proportion	Censoring rates			
		0.1	0.3	0.5	0.7
$x_k = 0.5x_l$	0	0.035	0.042	0.040	0.052
	20%	0.048	0.048	0.042	0.057
	30%	0.048	0.051	0.052	0.047
$x_k = sin^2 x_l$	0	0.049	0.052	0.050	0.049
	20%	0.048	0.044	0.044	0.055
	30%	0.047	0.045	0.049	0.045

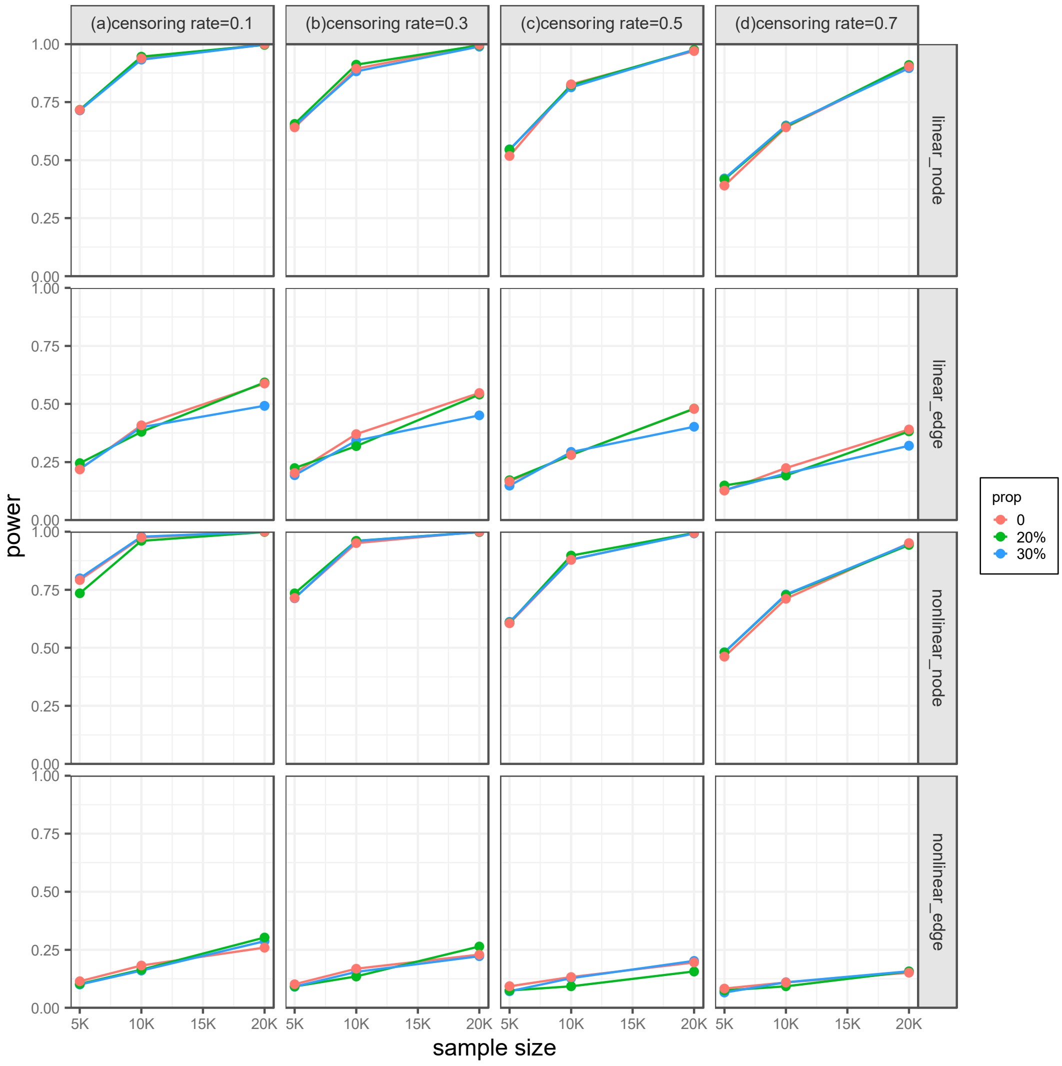

Figure S11. Power of CoNet in a reduced gene network with some genes being unavailable, where the effecting nodes and effecting edges are randomly selected. Power (y-axis) at a significance level 0.05 is plotted against different sample size (x-axis). The proportions of "20%" and "30%" represent 20% and 30% genes are unavailable in network, "0" means the genes are all available. Various settings are displayed from top to bottom, including the power for detecting the effect of node and the effect of edge in linear relationship as well as in nonlinear (the recombination of quadratic and sine relationship) relationship. Different censoring rates are listed from left to right, including (**a**) 0.1, (**b**) 0.3, (**c**) 0.5 and (**d**) 0.7.

Table S32. Type I error of CoNet and TIGAR to test the effect of nodes under the survival phenotype. Simulations were conducted with four different between-node correlation patterns (the combination of sine and quadratic, sine, quadratic, and linear) and three different censoring rates (0.1, 0.3, and 0.5).

Correlation patterns	Methods	Censoring rate		
**sample size=5000**				
		0.1	0.3	0.5
$x_k = 0.5x_l$	CoNet	0.051	0.050	0.046
	TIGAR	0.058	0.048	0.059
$x_k = sinx_l$	CoNet	0.037	0.047	0.043
	TIGAR	0.038	0.042	0.048
**sample size=10000**				
Correlation patterns	Methods	Censoring rate		
		0.1	0.3	0.5
$x_k = 0.5x_l$	CoNet	0.048	0.048	0.046
	TIGAR	0.045	0.050	0.046
$x_k = sinx_l$	CoNet	0.054	0.053	0.057
	TIGAR	0.059	0.048	0.053
**sample size=20000**				
Correlation patterns	Methods	Censoring rate		
		0.1	0.3	0.5
$x_k = 0.5x_l$	CoNet	0.054	0.060	0.051
	TIGAR	0.036	0.052	0.038
$x_k = sinx_l$	CoNet	0.050	0.053	0.055
	TIGAR	0.059	0.049	0.052

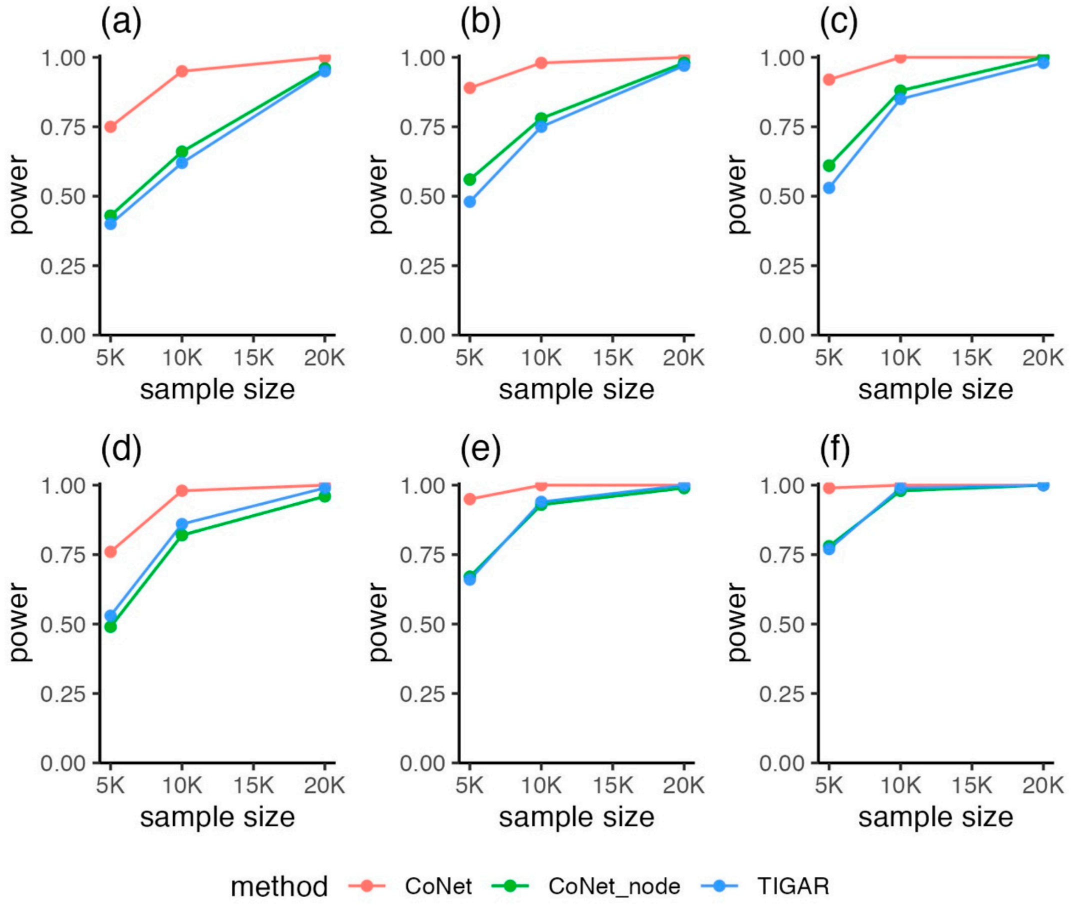

Figure S12. Power of CoNet and TIGAR to test the effect of nodes under the effect size of between-node correlation being 0.1. Power (y-axis) at a significance level 0.05 is plotted against different sample size (x-axis). "CoNet" and "CoNet_node" represent that CoNet tests the entire network and CoNet only tests nodes, respectively. The power with linear between-node correlation is shown under censoring rate 0.5 (**a**), 0.3 (**b**) and 0.1(**c**). The power with nonlinear between-node correlation (sine relationship) is shown under censoring rate 0.5 (**d**), 0.3 (**e**) and 0.1 (**f**).
